# OncomiR Addiction Is Generated by a miR-155 Feedback Loop in *Theileria*-Transformed Leukocytes

**DOI:** 10.1371/journal.ppat.1003222

**Published:** 2013-04-18

**Authors:** Justine Marsolier, Sandra Pineau, Souhila Medjkane, Martine Perichon, Qinyan Yin, Erik Flemington, Matthew D. Weitzman, Jonathan B. Weitzman

**Affiliations:** 1 Université Paris Diderot, Sorbonne Paris Cité, Epigenetics and Cell Fate, UMR 7216 CNRS, Paris, France; 2 Tulane Health Sciences Center, Tulane Cancer Centre, New Orleans, Louisiana, United States of America; 3 Department of Pathology and Laboratory Medicine, University of Pennsylvania Perelman Medical School and Children's Hospital of Philadelphia, Philadelphia, Pennsylvania, United States of America; Yale University, United States of America

## Abstract

The intracellular parasite *Theileria* is the only eukaryote known to transform its mammalian host cells. We investigated the host mechanisms involved in parasite-induced transformation phenotypes. Tumour progression is a multistep process, yet ‘oncogene addiction’ implies that cancer cell growth and survival can be impaired by inactivating a single gene, offering a rationale for targeted molecular therapies. Furthermore, feedback loops often act as key regulatory hubs in tumorigenesis. We searched for microRNAs involved in addiction to regulatory loops in leukocytes infected with *Theileria* parasites. We show that *Theileria* transformation involves induction of the host bovine oncomiR miR-155, via the c-Jun transcription factor and AP-1 activity. We identified a novel miR-155 target, DET1, an evolutionarily-conserved factor involved in c-Jun ubiquitination. We show that miR-155 expression led to repression of DET1 protein, causing stabilization of c-Jun and driving the promoter activity of the *BIC* transcript containing miR-155. This positive feedback loop is critical to maintain the growth and survival of *Theileria*-infected leukocytes; transformation is reversed by inhibiting AP-1 activity or miR-155 expression. This is the first demonstration that *Theileria* parasites induce the expression of host non-coding RNAs and highlights the importance of a novel feedback loop in maintaining the proliferative phenotypes induced upon parasite infection. Hence, parasite infection drives epigenetic rewiring of the regulatory circuitry of host leukocytes, placing miR-155 at the crossroads between infection, regulatory circuits and transformation.

## Author Summary

*Theileria* is the only intracellular eukaryotic parasite known to transform its host cell into a cancer-like state. Infection by the *T. annulata* parasite causes tropical theileriosis, killing large numbers of cattle in North Africa and Asia, and the related *T. parva* parasite causes East Coast Fever. We investigated whether transformation of host bovine leukocytes was associated with deregulation of small, non-coding RNAs. We discovered that transformation by *Theileria* leads to upregulation of an oncogenic small RNA called miR-155 which is contained within the *BIC* gene. Parasite induction of the microRNA involves activation of the transcription factor c-Jun which controls the *BIC* gene promoter. We identified a new target for the miR-155; the DET1 protein which is responsible for degradation of the c-Jun factor. This leads to a regulatory feedback loop that is critical for the transformed phenotype of the infected cells. We show that miR-155 expression inhibits DET1 protein translation, leading to accumulation of c-Jun protein and activation of the *BIC* gene containing miR-155. This is the first study to report regulation of oncogenic non-coding RNAs by *Theileria* and the novel feedback loop underlying the parasite-induced transformation.

## Introduction

Both infection and cancer have been extensively linked to the induction of microRNAs (miRs) which can exert diverse effects on cellular phenotypes by targeting many genes [1], [2]. microRNAs (miRNAs) are a class of small non-coding RNAs, 22 nt in length, that modulate post-transcriptional gene expression [1]. It is likely that miRNAs play critical roles in fine-tuning the host response to infection and inflammation [1], [3]. OncomiRs are miRNAs that are upregulated in tumours and which have oncogenic functions depending on the genes they target [4], [5]. However, It has been relatively difficult to identify essential miR pathways in infection and critical OncomiR target genes in tumorigenesis [6], [7]. ‘Oncogene addiction’ is an emerging concept which suggests that underlying the multistep nature of tumour progression, cancer cell growth and survival can often be impaired by targeting a single oncogene pathway, thereby offering a promise for the development of targeted molecular therapies [8], [9], [10].

To investigate whether microRNAs could link infection to tumorigenesis, we studied a unique model of reversible transformation induced following infection by an intracellular parasite. The lymphoproliferative disease induced by the intracellular protozoan parasite *Theileria* constitutes a powerful model system to explore the signaling and epigenetic mechanisms underlying transformed phenotypes [11], [12], [13]. The tick-transmitted parasites *T. annulata* and *T. parva* infect bovine leukocytes leading to proliferative and invasive phenotypes which partially mirror lymphoma pathologies when injected into immunocompromised mice [12], [14], [15]. *Theileria*-infection causes hyperproliferation, invasiveness and escape from apoptosis, presumably through the manipulation of host cellular pathways [16], [17]. Several host signaling mechanisms have been implicated, including c-Jun N-terminal Kinase (JNK) and host nuclear factors c-Myc, NFκB and AP-1 [16], [18], [19], [20], [21], but the transcriptional networks regulated by these factors are not fully defined. Furthermore, the transformed phenotypes of *Theileria*-infected cells are curable by treatment with the theilericidal drug Buparvaquone (BW720c), which kills the parasite without any apparent toxicity towards host cells [13], [18]. This led us to investigate whether oncogene addiction pathways and epigenetic switches contribute to transformation in these cells. We studied TBL3 cells which were derived by *in vitro* infection with *T. annulata* of BL3 cells, an immortalized, bovine B lymphocyte cell line. Specifically, we investigated whether the transformed phenotype of the *Theilieria*-infected cells is associated with deregulation of miRNA pathways. miRNA networks are affected by several parasites of the apicomplexa phylum (e.g. *Toxoplasma* [22], *Cryptosporidium* [23] or *Eimeria* [24]). However, *Theileria* offers a particularly interesting study model because of its unique ability to transform host leukocytes.

The oncomir miR-155 is one of the best studied oncogenic miRNAs and it has been extensively linked to inflammation, induced by a range of bacterial pathogens and viruses [25], [26], [27]. miR-155 resides in a non-coding transcript, called *BIC*, first identified in chickens as a site of retroviral insertion in avian leukosis virus-induced lymphomas [28], [29]. Homologues of *BIC* (B-cell integration cluster) have been identified in humans and mice and contain the precursor hairpin encoding miR-155. *BIC* and miR-155 are overexpressed in lymphomas, including Hodgkin's lymphoma, and acute myeloid leukemia patients, as well as several solid tumours [26], [27]. The promoter of the *BIC* gene contains a highly conserved recognition motif for the transcription factor AP-1 formed by heterodimers of Jun and Fos proteins [30]. Transgenic mice overexpressing miR-155 in B cells developed lymphoproliferative disorders, whereas knockout mice have also demonstrated that miR-155 plays a critical role in the development of the immune system and the adaptive immune response [31], [32]. The mechanisms by which the oncomiR-155 drives and maintains tumorigenesis remain relatively unclear and few molecular targets have been identified that explain miR-155 contribution to inflammation or the cancer cell phenotypes.

Here we show that *miR-155* and *BIC* upregulation are features of cells infected by the parasite *Theileria*. We identified AP-1/Jun as a transcriptional regulator of *BIC* in these cells. We also identified a new miR-155 target, transcripts encoding the DET1 protein which is involved in targeting c-Jun for degradation by ubiquitination. Thus, miR-155 expression leads to DET1 down-regulation, accumulation of the c-Jun protein and activation of the *BIC* promoter. This feedback loop is essential for miR-155 oncogenic function and, thus, *Theileria* infection of the host leukocytes creates a transformed state involving addiction to both parasite and oncomiR.

## Results

### miR-155 is upregulated in bovine leukocytes transformed by *Theileria* infection

**Figure 1:**
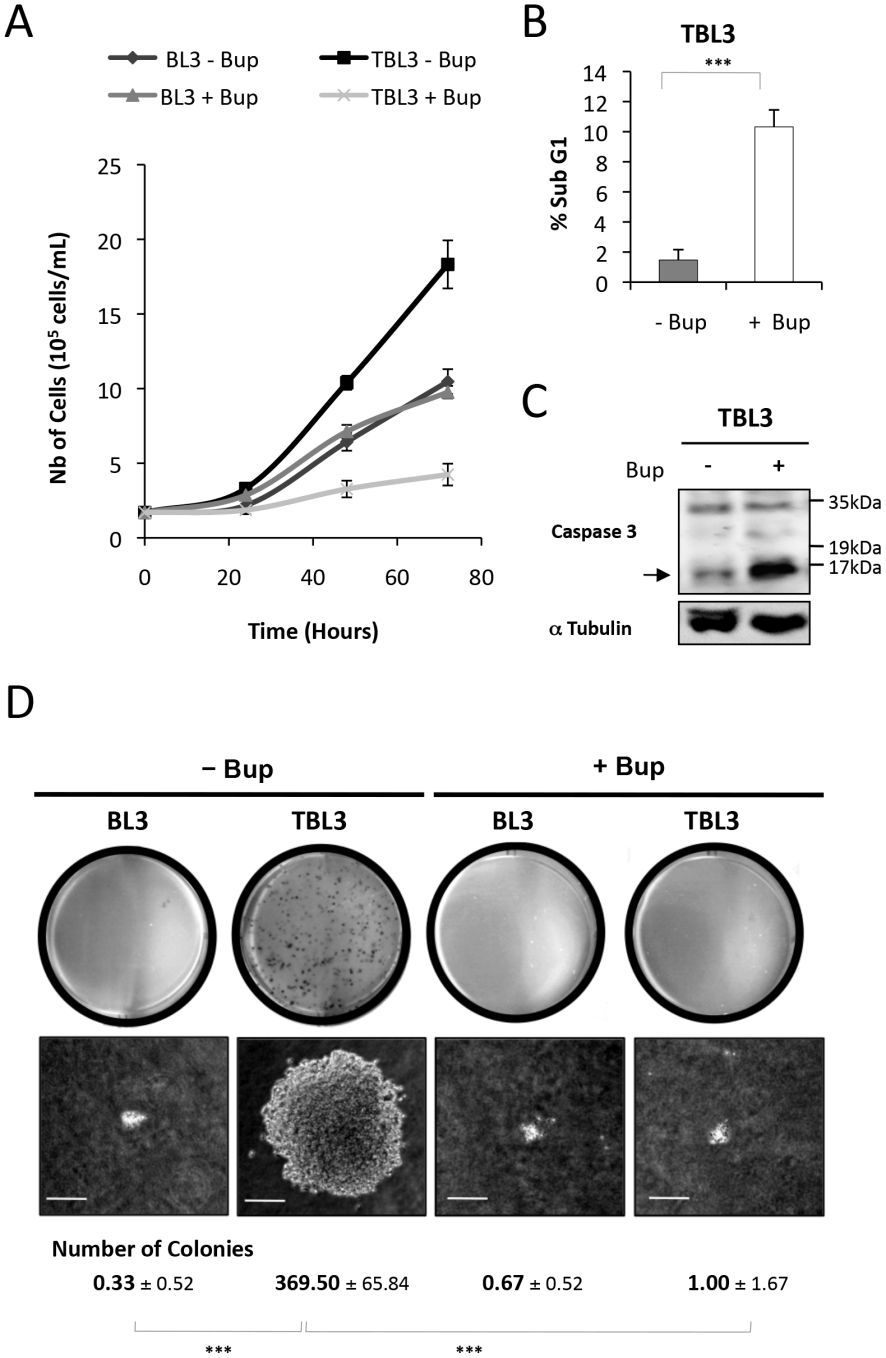
*Theileria* is required to maintain the transformed phenotype of parasitized cells. **(A)** The parasite-infected TBL3 cells were grown in the presence or absence of Buparvaquone (+Bup) and cell numbers were monitored by counting live cells following trypan blue exclusion. Buparvaquone had no effect on the proliferation of non-parasitized BL3 cells (average ± standard deviation, n = 3). **(B)** Representation of flow cytometry analysis indicating the induction of apoptosis (sub-G1 population) in TBL3 cells following treatment with Buparvaquone (open histograms - average ± sd, n = 3). **(C)** Inhibiting *Theileria* with Buparvaquone caused apoptosis as monitored by Western blot detection of Caspase-3 activation. The arrow indicates cleaved activated Caspase-3 (n = 3). **(D)** Parasite-infected TBL3 cells formed colonies when grown in soft agar, in contrast to uninfected parental BL3 cells. This transformed phenotype was reversed by incubating with Buparvaquone (+Bup). The number of colonies per plate (average ± sd, n = 3) and their appearance under the microscope 10 days after plating are shown. (average ± sd, n = 3). ***p<0.001.

To investigate the molecular mechanisms underlying the phenotypes of *Theileria*-infected cells, we studied TBL3 cells which were derived by *in vitro* infection with *T. annulata* of BL3 cells, an immortalized, bovine B lymphocyte cell line. Treatment with the theilericidal drug Buparvaquone caused reduced proliferation in TBL3 cells, while it had no effect on the growth of the parental BL3 cells (Figure 1A). The TBL3 growth arrest was due to reduced cell cycle progression (as measured by Ki67 labeling) (Figure S1B) and apoptosis in these cells (as measured by flow cytometry and caspase activation) (Figure 1B and 1C). The parasitized TBL3 cells have constitutive AP-1 activation [20] and formed colonies when grown in soft agar, which was also reversed by Buparvaquone treatment (Figure 1D). We observed similar effects of Buparvaquone on Thei cells, a naturally infected macrophage cell line derived from a tumour of an infected cow (Figure S1A). Thus, *Theileria*-infected cells are ‘addicted’ to the presence of live parasites which is necessary for maintaining growth and survival.

**Figure 2:**
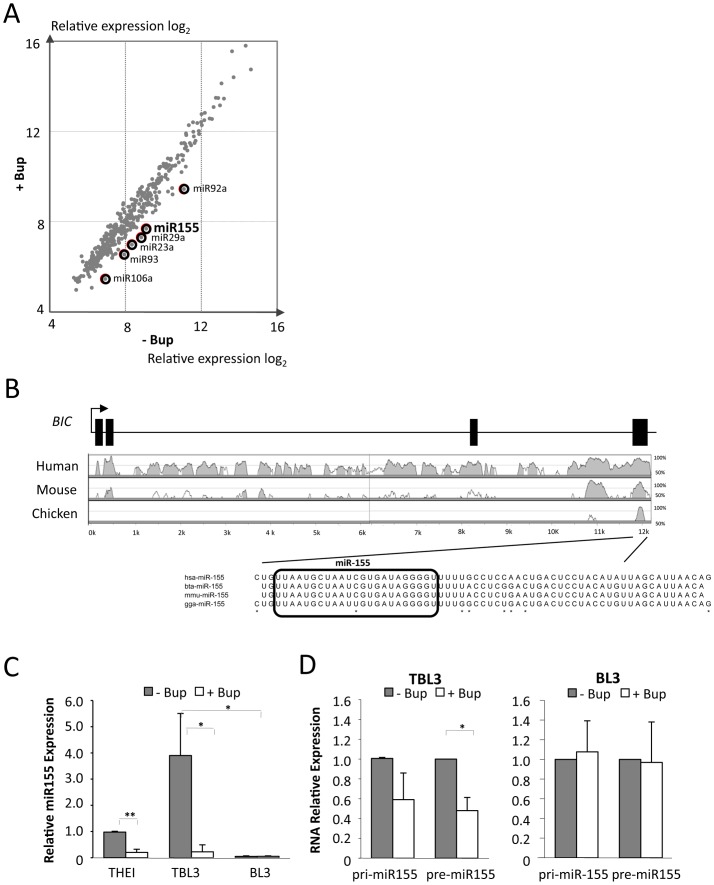
The oncomiR miR-155 is upregulated in cells transformed by *Theileria*. **(A)** We performed miRNA microarray analysis using RNA from parasitized TBL3 cells, treated or not with Buparvaquone for 64 hours. The scatter blot represents the expression of all 459 miRNA data points. Six miRs (highlighted by circles) were down-regulated by more that 2.5-fold (log2) upon Buparvaquone treatment. **(B)** The Vista plot shows conservation of the *BIC* gene, comparing bovine to human, mouse or chicken sequences. The miR-155 sequence is identical in the bovine (Bta), human (Hsa) and chicken (Gga) genomes. The square boxes represent the positions of the exons in the human *BIC* gene. Black arrow represents the Transcriptional start site (TSS). **(C)** Analysis of miR-155 expression by TaqMan qPCR comparing RNA isolated from Thei, TBL3 or BL3 cells in the presence or absence of Buparvaquone (Bup). Transcript levels in untreated cells are shown relative to the control and normalized against RNU6B mRNA (average ± sd, n = 3). **(D)** Relative RNA levels of immature pri-miR-155 or pre-miR-155 transcripts in parasitized TBL3 or parental BL3 cells in the presence or absence of Buparvaquone (Bup). Transcript levels in untreated cells are shown relative to the control and normalized against β-actin and B2M mRNA (average ± sd, n = 3). *p<0.05, **p<0.01, ***p<0.001.

Infection is increasingly linked to the induction of microRNAs which can exert diverse effects on host cellular phenotypes by targeting many genes [1], [3]. We hypothesized that miRs could play a role in *Theileria*-induced transformation and that the presence of active parasites could induce oncomiR expression. We examined the expression of host microRNAs in parasitized TBL3, with or without Buparvaquone treatment, by microarray analysis (Figure 2A). We found that six miRs were consistently down-regulated more than 2-fold in the *Theileria-*infected cells following Buparvaquone treatment (Figure 2A and Table S1). Several of these microRNAs have been linked to tumorigenesis and human leukemia (Table S1). We focused on the miR-155 oncomiR for a number of reasons; miR-155 was lowly expressed in the parental BL3 cell line (see below) and miR-155 is overexpressed in human B cell lymphoma, leukemia, breast cancer and pancreatic cancer [27], [33]. miR-155 was also shown to cause cancer in genetically engineered mice and has been extensively linked to infection and inflammation [31], [34], [35]. The miR-155-containing *BIC* gene, was originally identified as a common site for insertion of proviral DNA in avian virus-induced lymphomas [28], [29] and is induced in human lymphomas transformed by Epstein-Barr Virus (EBV) [30], [36], [37]. These results suggested that miR-155 could be a common target used by viruses and parasites to manipulate host cell functions during cancer and infection.

Comparative genomic analysis revealed that the *BIC* gene is conserved across species and that the mature miR-155 sequence is identical between human and bovine genomes [38] (Figure 2B). We confirmed the miRNA microarray data by TaqMan quantitative PCR (qPCR) analysis. miR-155 was significantly upregulated in TBL3 cells compared to the non-parasitized parental BL3 cells (Figure 2C). Furthermore, Buparvaquone treatment caused a dramatic decrease in miR-155 expression in both TBL3 lymphocytes and Thei macrophage cell lines (Figure 2C). We also tested the expression of immature transcripts pri-miR-155 and pre-miR-155 by qPCR; we observed that these forms were reduced by Buparvaquone in TBL3 cells, but not in non-infected BL3 cells (Figure 2D).

**Figure 3:**
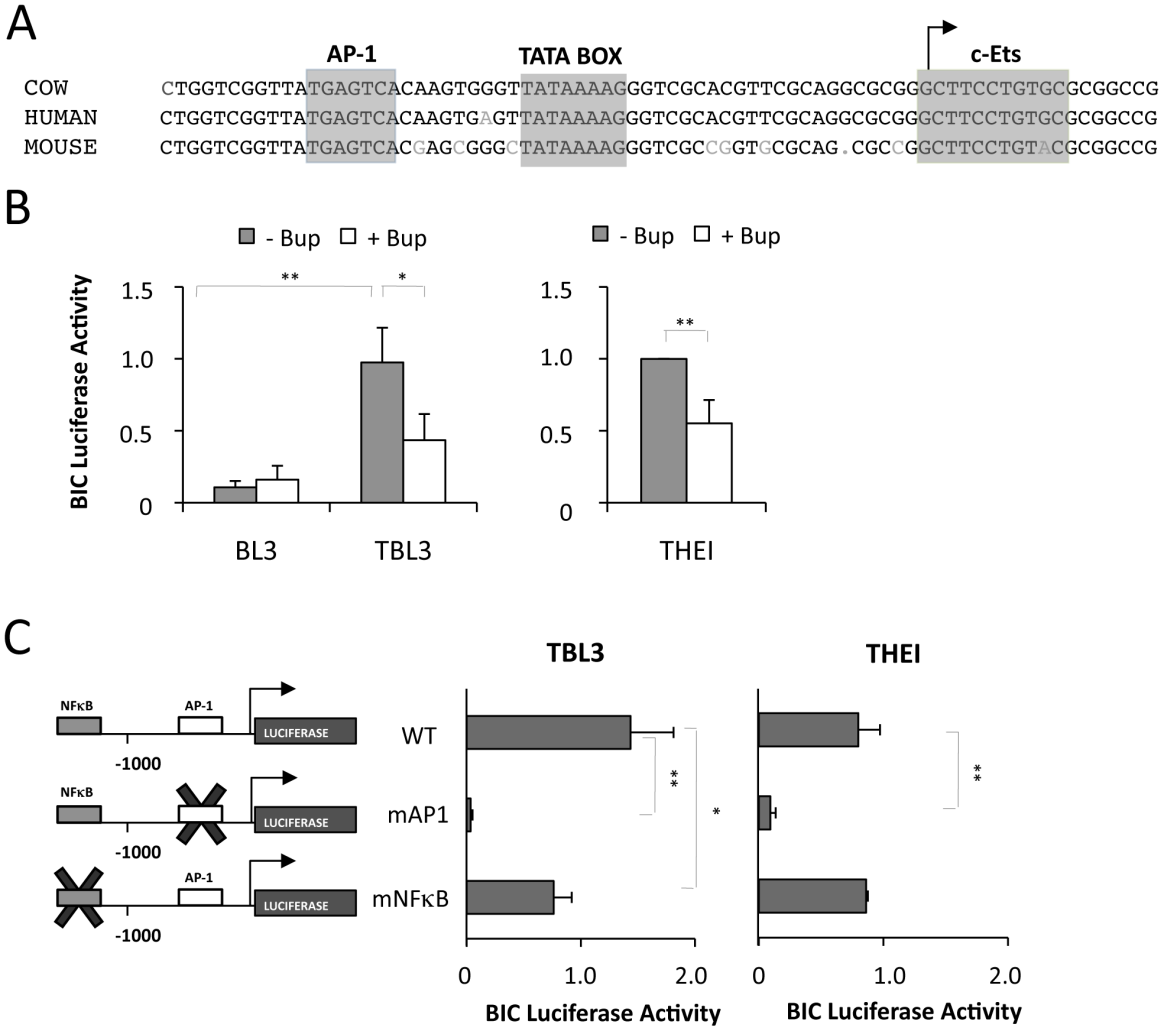
The transcriptional induction of *miR-155* in *Theileria* infected-cells is dependent on AP-1. **(A)** Sequence alignment of the proximal promoter sequence of the bovine, human and mouse *BIC* genes. The highly conserved AP-1 sites and TATA box are highlighted. The TSS is indicated by an arrow. **(B)** Luciferase reporter analysis of *BIC* promoter activity in cells infected with *Theileria* (TBL3 or THEI) or non-infected BL3 cells, with or without Buparvaquone treatment. All experiments were normalized by co-transfection with Renilla constructs (average ± sd, n = 3). **(C)** The effect of mutating AP-1 (mAP1) or NFκB (mNFκB) binding sites in *BIC* promoter luciferase assays was tested in TBL3 or THEI cells (average ± sd, n = 3). *p<0.05, **p<0.01.

The human *BIC* gene was shown to be transcriptionally regulated by the AP-1 transcription factor in EBV-transformed lymphomas [30]. Moreover, *Theileria* transformation is characterized by constitutive AP-1 activation [19], [20]. We therefore investigated whether *miR-155/BIC* induction in *Theileria*-infected cells is dependent on AP-1/Jun. Sequence alignments showed that the AP-1 binding site in the proximal *BIC* promoter is highly conserved across species (Figure 3A). To test whether *BIC* is transcriptionally regulated by *Theileria*, we transfected cells with a reporter construct containing the *BIC* promoter driving the luciferase gene (*BIC-Luc*). The activity of the *BIC* promoter was significantly higher in TBL3 cells compared to non-infected BL3 cells (Figure 3B). Moreover, the *BIC* promoter activity in infected cells was decreased by treatment with Buparvaquone (Figure 3B). The activity of the *BIC-Luc* reporter was also decreased by Buparvaquone treatment in Thei cells (Figure 3B). To test the involvement of AP-1 and NFκB transcription factors in *BIC* expression in these cells, we used promoter constructs mutated within the AP-1 or NFκB binding sites [30]. We observed that mutation of the conserved AP-1 binding site dramatically reduced *BIC* promoter activity in both cell lines, whereas mutation of the NFκB binding site had less effect (Figure 3C). Therefore, we conclude that *Theileria* regulates miR-155 primarily by AP-1 driven transcription of the bovine *BIC* gene.

### DET1 is a novel miR-155 target

**Figure 4:**
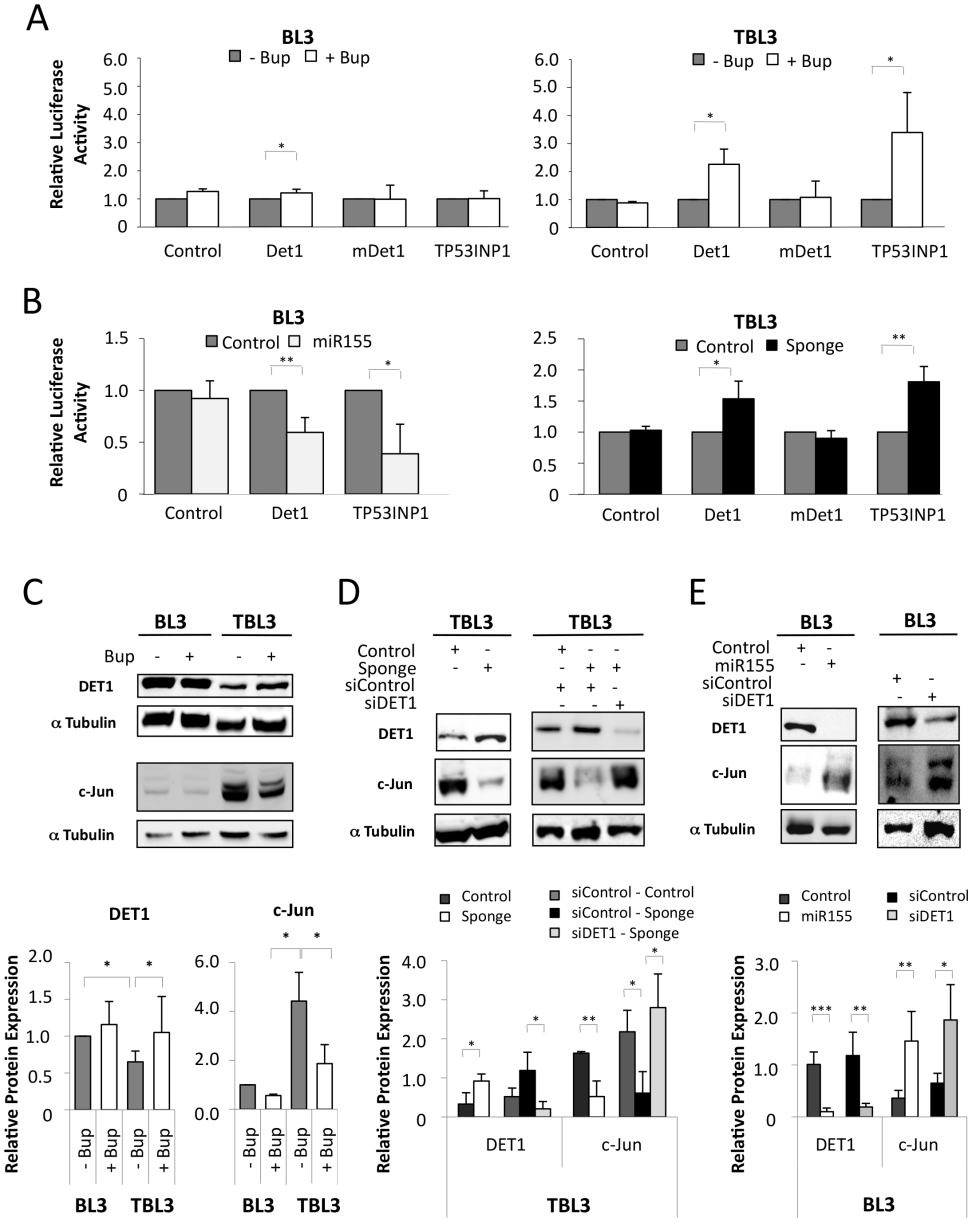
Bovine DET1 is a direct target of the miR-155 oncomiR. **(A)** Luciferase reporters containing the total 3′UTR of DET1 and TP53INP1 show that Buparvaquone induced Luciferase in parasitized TBL3 cells, but not in parental BL3 cells, and Luciferase induction was lost when the miR-155 target binding site was mutated (mDet1) (average ± sd, n = 3). **(B)** Luciferase-3′UTR analysis demonstrated that the DET1 and TP53INP1 reporters were suppressed by miR-155 transfection in BL3 cells (left panel) and that a miR-155 Sponge inhibitor induced luciferase activity in TBL3 cells (right panel), dependent on the miR-155 target site (average ± sd, n = 3). **(C)** Western blot analysis of DET1 and its target protein c-Jun. TBL3 cells showed reduced DET1 protein and elevated c-Jun compared to unparasitized BL3 cells, and the levels were reversed by treatment with Buparvaquone. α Tubulin was used as a loading control. Relative quantification (lower panel) indicates the DET1/Tubulin and c-Jun/Tubulin ratios calculated with Image J software (average ± sd, n = 3). **(D)** Inhibition by the miR-155 Sponge in TBL3 cells caused upregulation of DET1 protein and decreased c-Jun protein. These effects were reversed by siRNA against DET1. Relative quantification (lower panel) indicates the DET1/Tubulin and c-Jun/Tubulin ratios calculated with Image J software (average ± sd, n = 3). **(E)** In BL3 cells, the transfection of either miR-155 or siRNA DET1 caused decreased DET1 protein and elevated c-Jun protein levels. Relative quantification (lower panel) indicates the DET1/Tubulin and c-Jun/Tubulin ratios calculated with Image J software (average ± sd, n = 3). *p<0.05, **p<0.01, ***p<0.001.

To understand the contribution of upregulated miRs to cellular phenotypes, it is important to identify functionally-relevant targets whose expression is regulated by miR action. To identify putative miR-155 target genes, we performed a computational screen for genes with complementary miR-155 sites in their 3′UTR using online software (including Microcosm targets, TargetScan and PicTar). We found that *DET1*, *JARID2* and *TP53INP1* genes are putative miR-155 targets; they exhibit a strong seed match and the binding site is conserved across species (Supplementary Figure S2A). We performed qPCR analysis to investigate the mRNA level of these genes in infected leukocytes, but found no significant difference between the expression of these genes in TBL3 or Thei cells upon Buparvaquone treatment (Supplementary Figure S2C). Consequently, we tested whether the effect of miR-155 on these potential targets could occur via inhibition of translation. We transfected luciferase reporters fused to the miR-155-targeted 3′UTR of these genes into *Theileria*-infected cells and tested the effect of Buparvaquone [30]. The relative activities of 3′UTR-Luciferase constructs were significantly increased by Buparvaquone treatment in TBL3 cells and in Thei cells, but not in BL3 cells (Figure 4A and Supplementary Figure S2B). We focused on one of these potential targets; DET1, a highly conserved protein reported to promote the ubiquitination and degradation of the proto-oncogenic transcription factor c-Jun [39]. Mutation of the miR-155 target site in the DET1 Luciferase-3′UTR reporter (mDET1) abolished the Buparvaquone-induced luciferase activity in TBL3 cells (Figure 4A). We used TP53INP1, a pro-apoptotic tumour suppressor protein reported to be repressed by miR-155 in pancreatic tumours [40], as a positive control (Figure 4A). The effects of Buparvaquone could include changes in many miRs, so we performed experiments in BL3 cells in which we cotransfected either the DET1 or the TP53INP1 Luciferase-3′UTR reporters with a miR-155-expressing plasmid. The DET1 and TP53INP1 3′UTR-reporters were inhibited by co-transfection with miR-155, but not control (Figure 4B). Conversely, we performed a series of experiments involving co-transfection with a miR-155 “Sponge” construct, which functions as a miR-155 inhibitor. The Sponge inhibitor increased expression of the DET1 and TP53INP1 3′UTR-reporters, but not the mutated DET1 construct (Figure 4B).

**Figure 5:**
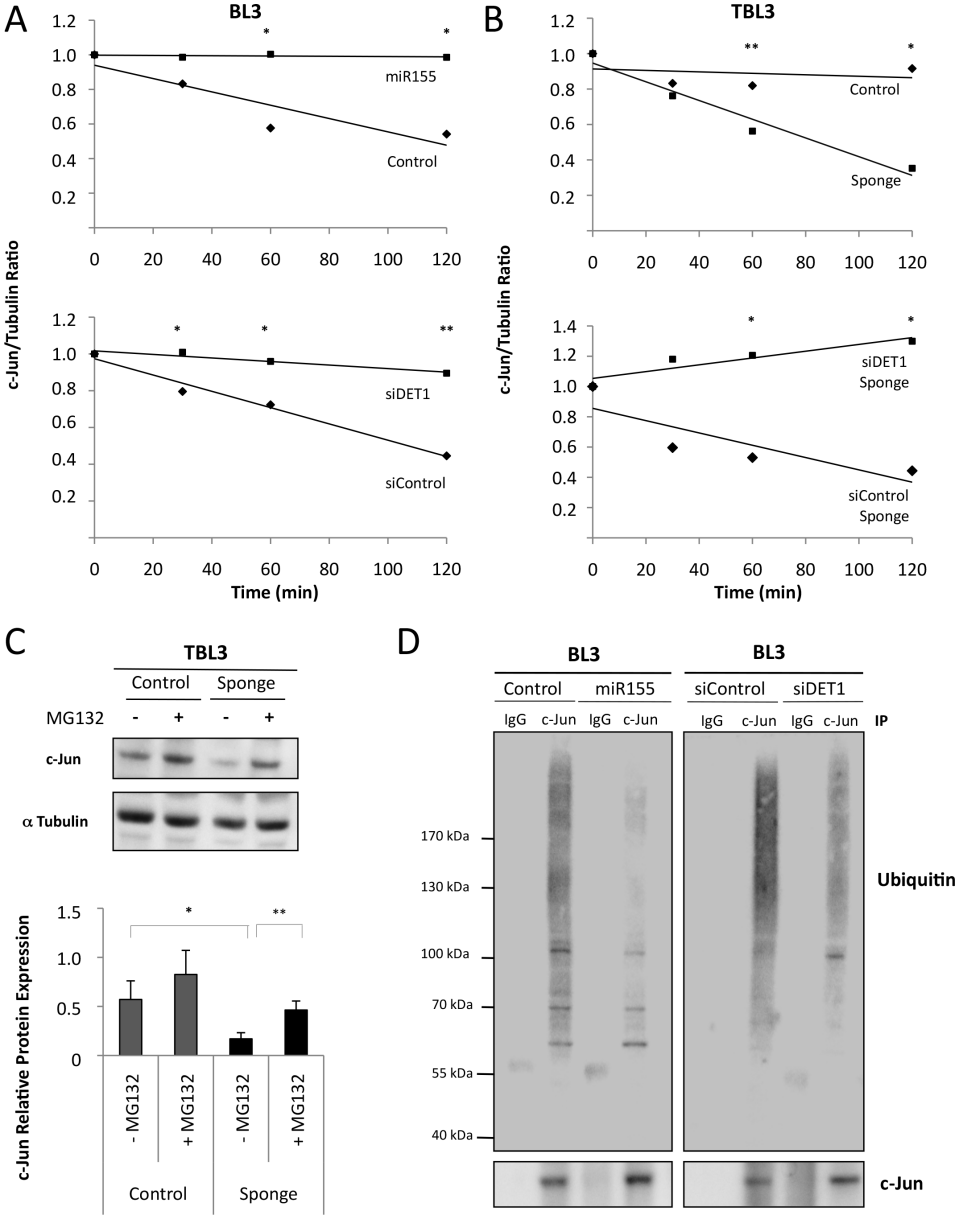
miR-155 stabilized c-Jun by inhibiting its proteasomal degradation. **(A)** Overexpression of miR-155 or depletion of DET1 in BL3 cells increased the half-life of endogenous c-Jun protein. BL3 cells transiently expressing miR-155 or siDET1 were treated with cycloheximide for the indicated times, followed by immunoblot analysis with a c-Jun antibody and semi-quantification with an αTubulin antibody as a loading control. Relative c-Jun protein levels at time 0 were set as 1 (average ± sd, n = 3). **(B)** Inhibition by the miR-155 Sponge in TBL3 cells decreased the half-life of endogenous c-Jun. These effects were reversed by siRNA against DET1. TBL3 cells transiently expressing miR-155 Sponge +/− siDET1 were treated with cycloheximide for the indicated times, followed by immunoblot analysis with a c-Jun antibody and semiquantification with α-Tubulin as a loading control. Relative c-Jun levels at time 0 were set as 1 (average ± sd, n = 3). **(C)** Effect of the miR-155 Sponge on c-Jun protein levels was rescued by treating the proteasome inhibitor MG132. TBL3 cells transiently expressing the miR-155 Sponge were treated with MG132 for 3 h, followed by immunoblot analysis with the c-Jun antibody and semiquantification with α Tubulin as a loading control (average ± sd, n = 3). **(D)** Overexpression of miR-155 or depletion of DET1 in BL3 cells reduced c-Jun ubiquitination. Transfected cells were treated with MG132 for 3 h, followed by endogenous c-Jun immunoprecipitation and immunoblot analysis with indicated antibodies (average ± sd, n = 3). *p<0.05, **p<0.01, ***p<0.001.

These results suggest that DET1 protein translation is directly targeted by miR-155 binding to the 3′UTR sequence. To confirm this at the protein level, we performed Western blot analysis; DET1 levels were reduced in TBL3 cells compared to BL3 cells (Figure 4C). In TBL3 cells, treatment with Buparvaquone or transfection with the miR-155 Sponge inhibitor both resulted in elevated DET1 protein, and decreased c-Jun levels (Figure 4C and 4D). Furthermore, the effect of the miR-155 Sponge is DET1-dependent, as it was reversed by siRNA specifically targeting DET1 expression (Figure 4D). Conversely, transfecting miR-155 into BL3 cells reduced DET1 levels and led to elevated c-Jun protein(Figure 4E). This could be mimicked by transfecting with siRNA against DET1 (Figure 4E). Although DET1 regulates c-Jun degradation by the ubiquitin-dependent proteosome [39], DET1 was recently shown to participate in transcriptional repression in plants [41]. To confirm that the miR-155 levels and DET1 targeting affected c-Jun protein stability in our cells, rather than transcription, we investigated c-Jun stability by pulse-chase labeling with cycloheximide (Figure 5A and 5B). We showed that both miR-155 and siDET1 decreased c-Jun degradation in BL3 cells (Figure 5A). Conversely, the miR-155 Sponge enhanced c-Jun degradation in infected TBL3 cells and this was rescued by siDET1 (Figure 5B). Additional experiments using the MG132 proteosome inhibitor, confirmed that c-Jun inhibition by the miR-155 Sponge in TBL3 cells was due to proteosome-dependent degradation (Figure 5C). Analysis of the c-Jun mRNA levels by qPCR also confirmed that the effects of miR-155, siDET1 and the miR-155 Sponge are at the protein level without changes in c-Jun transcripts (Supplementary Figure S3A and S3B). Finally, we looked at c-Jun ubiquitination in our cells; transfection with either miR-155 or siDET1 decreased c-Jun ubiquitination in BL3 cells, consistent elevated c-Jun stability (Figure 5D). In contrast, c-Jun ubiquination levels were higher in TBL3 cells transfected with the miR-155 Sponge (Supplementary Figure S3C). Together, these experiments show that miR-155 can target DET1 leading to c-Jun accumulation in transformed *Theileria-*infected leukocytes.

### miR-155 participates in an addictive feedback loop

**Figure 6:**
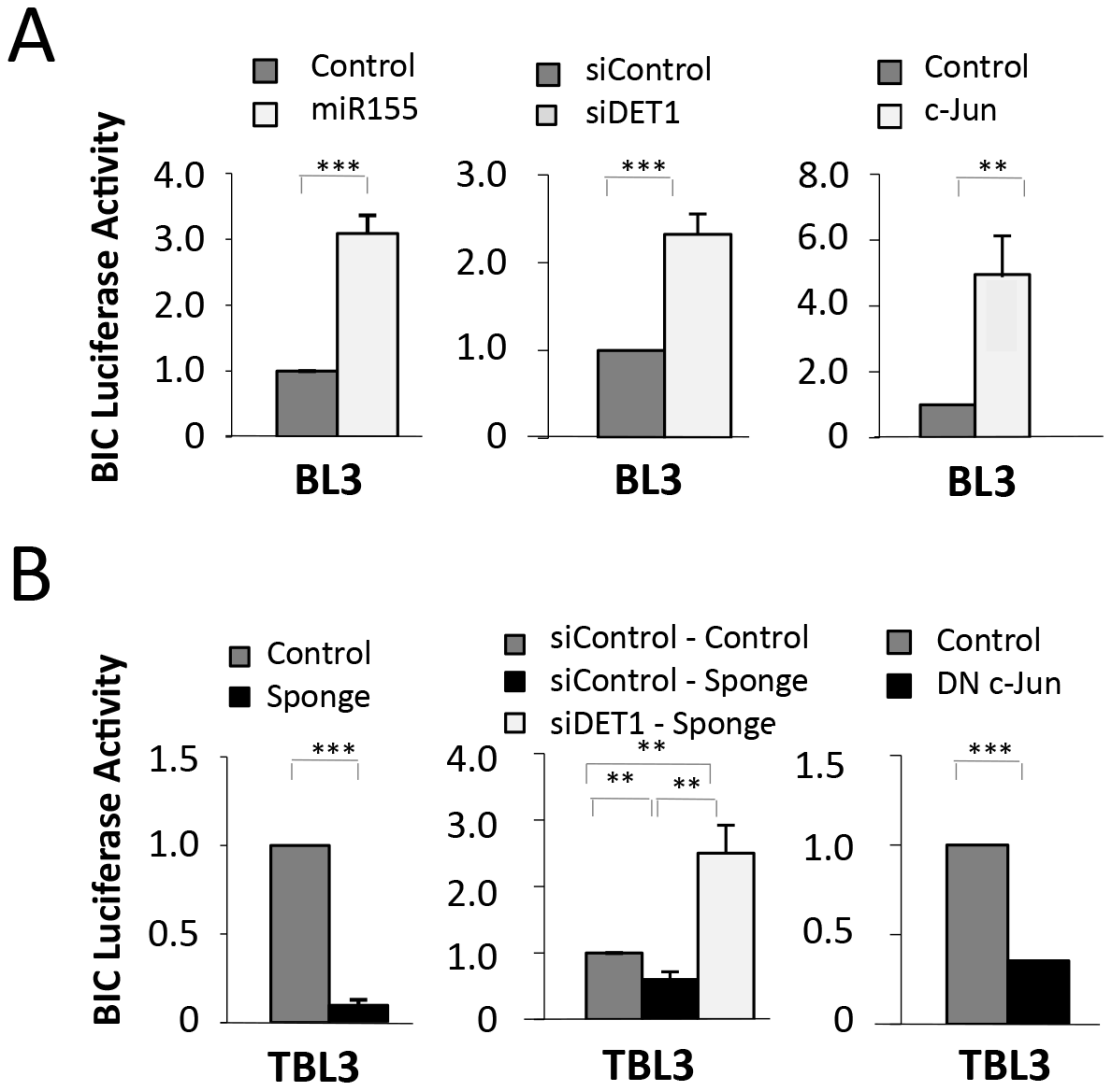
miR-155 drives a feedback regulatory loop. **(A)** miR-155 directly regulates the BIC promoter. In unparasitized BL3 cells, transfection with miR-155, siDET1 or c-Jun led to induction of the *BIC-Luciferase* reporter. Relative luciferase results are shown compared to control transfections in BL3 cells (dark grey bars) (average ± sd, n = 3). **(B)** In contrast, in TBL3 cells, inhibiting miR-155 or c-Jun (with plasmids encoding miR-155 Sponge or Dominant Negative DN-c-Jun, respectively) led to reduced *BIC*-*Luciferase* reporter activity (average ± sd, n = 3). *p<0.05, **p<0.01, ***p<0.001.

As the expression of miR-155 led to reduced DET1 protein and elevated c-Jun levels, we hypothesized that this might increase AP-1 activity, thereby creating a positive feedback loop to drive expression of the *BIC* promoter. We tested this hypothesis in BL3 cells using the *BIC-Luc* reporter that we showed above was *Theileria*-regulated via AP-1 (Figure 6). We found that the expression of either miR-155 or siDET1 or c-Jun resulted in induction of *BIC* promoter activity in uninfected BL3 cells (Figure 6A). This suggested that upregulation of AP-1/c-Jun is sufficient to induce *BIC* expression in these cells and that miR-155 may induce the expression of its own promoter via AP-1/Jun activation. Conversely, we found that the *BIC-Luciferase* activity in TBL3 cells was reduced by co-transfection with the miR-155 inhibitory Sponge or a Dominant-Negative c-Jun (DN c-Jun) (Figure 6B). Finally, the inhibitory effect of the miR-155 Sponge on the *BIC* promoter could be overcome by suppressing DET1 using siRNA (Figure 6B, middle). These experiments suggested that the miR-155/DET1/Jun/BIC loop creates a regulatory feedback circuit.

**Figure 7:**
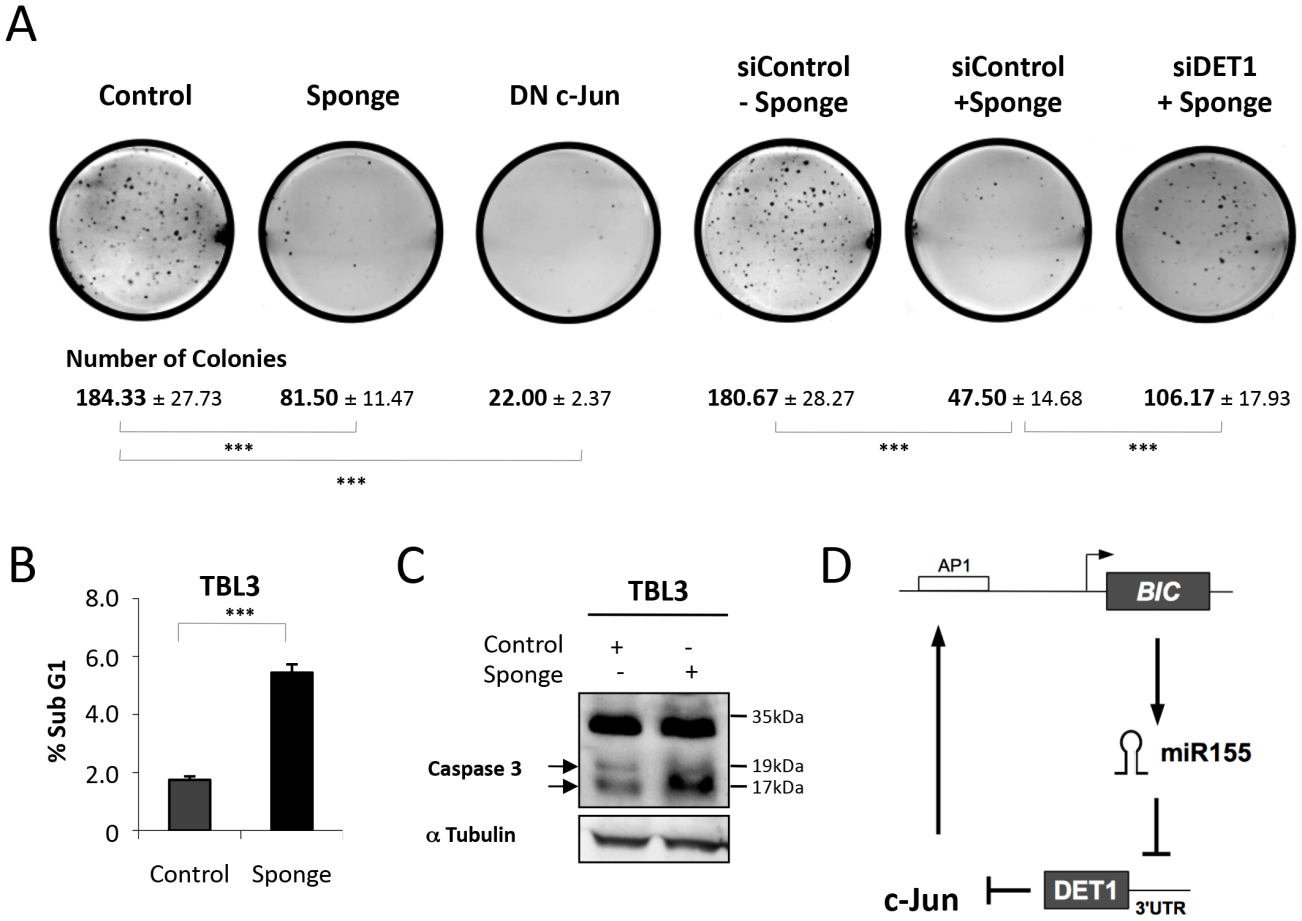
The miR-155/c-Jun loop is essential for growth and survival. **(A)** The colony forming potential of TBL3 cells was markedly reduced by transfection with miR-155 Sponge or Dominant Negative (DN) c-Jun. The inhibitory effect of the miR-155 Sponge could be reversed by co-transfection with siRNA inhibiting DET1, compared to a non-relevant scrambled siRNA control. The photographs are representative of three independent experiments and the average number of colonies per plate are indicated (average ± sd, n = 3). ***p<0.001 **(B)** Representation of flow cytometry analysis indicating the induction of apoptosis (sub-G1 population) of TBL3 expressed miR-155 Sponge (black histograms) (average ± sd, n = 3). **(C)** miR-155 expression is essential for TBL3 survival. Transfecting TBL3 cells with the inhibitory miR-155 Sponge caused apoptosis as monitored by Western blot detection of Caspase-3 activation. The arrows indicate cleaved activated Caspase-3 (n = 3). **(D)** A schematic representation of the miR-155/DET1/c-Jun regulatory loop driving oncomiR addiction. The positive feedback loop is provoked by miR-155 repression of DET1 protein translation and DET1-dependent repression of c-Jun protein stability.

To test the functional significance of this miR-155-DET1-Jun loop, we investigated the effect of blocking the hubs in the loop on the ability of transformed TBL3 cells to form colonies in the soft agar assay. Transfection of parasitized TBL3 cells with either the miR-155 Sponge or DN c-Jun caused a dramatic decrease in the number of colonies (Figure 7A). Notably, DN c-Jun has also been reported to reduce tumour formation by parasitized cells in mice experiments (19). The inhibitory effects of the Sponge were reversed by co-transfection with siDET1, but not control siRNA (Figure 7A). Thus, the regulatory loop seems to be essential for colony growth. Furthermore, we tested the effect of inhibiting the miR-155 loop on cell survival. The transfection of TBL3 cells with the miR-155 Sponge also caused significant apoptosis, as revealed by flow cytometry or Caspase-3 activation, equivalent to that induced by killing the parasite with Buparvaquone (Figure 7B and 7C). Thus, the miR-155 oncomiR loop is essential for parasite-induced host cell growth and survival, thereby creating a state of oncogene addiction (Figure 7D).

## Discussion

*Theileria*-induced transformation offers an attractive experimental model, as it appears that infection of host leukocytes is accompanied by a rewiring of the cellular circuitry [13], [17], [18]. The identification of molecular players that play key roles in maintaining proliferative phenotypes could be relevant for identifying effective therapeutic strategies to reverse transformation. Thus, oncogenic pathways in *Theileria*-infected cells may highlight examples of oncogene addiction for future studies. We have extended this hypothesis to investigate microRNA pathways and identified molecular targets that create an addictive regulatory loop. This is the first study to show that *Theileria* manipulates host gene expression via microRNAs. This observation underlines the increasing importance being given to non-coding RNAs in the regulation of gene expression, inflammatory response and tumour cell phenotypes [2], [3], [27]. miRNA networks are affected by several parasites of the apicomplexa phylum (e.g. *Toxoplasma* [22], *Cryptosporidium* [23] or *Eimeria* [24]). Some of these may be related to the infection process and initial inflammatory responses, while others may be relevant to long-term features of host-parasite interactions. *C. parvum* infection of epithelial cells was also shown to induce a range of host miRNAs which are regulated by NFκB-dependent transcription [23]. However, there does not seem to be any significant overlap with the miRNA network regulated by *Theileria*. Similarly, *T. gondii* was shown to induce transcriptional regulation of a distinct set of host miRNAs, whereas the related *Neospora caninum* parasite did not [22]. Future studies might reveal common and distinct pathways related to miRNA induction by parasites across the apicomplexa phylum. miR-155 induction does seem to be a common feature in several inflammatory and tumorigenic scenarios. For example, *Helicobacter pylori* infection, which is associated with gastric adenocarcinoma, also induces miR-155 expression in T cells, but via Foxp3 [42]. We show here that activated AP-1 transcription factors in parasitized transformed cells drives the transcription of the *BIC* gene, leading to increased miR-155 expression in both artificially infected bovine B cells and in naturally-infected bovine Thei macrophages. We provide evidence that miR-155 targets the DET1 protein, which leads to accumulation of the c-Jun protein and increased transcription of the miR-155-encoding *BIC* gene (Figure 7D). This feedback loop is critical for maintaining the transformed phenotype, as inhibiting any node in the loop reverses the transformed phenotypes (growth in soft agar and cell survival) of parasitized cells. Thus, our study has provided the molecular events in a miR-155 loop that links infection and transformation.

Host cell infection by *Theileria* parasites is accompanied by a range of signal transduction pathways including the IKK/NFκB and JNK/AP-1 pathways [16], [19], [20]. It is not clearly defined how these signaling pathways are integrated in the nucleus to drive gene expression programs that underlie the transformed phenotype. We found that AP-1 is critical for *BIC* promoter activity in both TBL3 lymphoctytes and Thei macrophages, whereas the contribution of NFκB, was relatively minor. It is possible that NFκB plays a role in *BIC* induction upon infection and that a epigenetic switch subsequently creates a dependence on the BIC/miR-155/c-Jun loop to maintain the transformed phenotype. Indeed, *Theileria*-infected cells can grow in immunocompromised mice [14], [15] and c-Jun was previously shown to be critical for *Theileria*-associated B cell growth *in vivo* [19]. Furthermore, *BIC* induction in B lymphocytes cause by infection with the Epstein-Barr Virus (EBV) is also driven by AP-1 activity [30]. These observations offer an interesting parallel between viruses and parasites in miRNA modulation during tumorigenic progression.

We report here that miR-155 represses DET1 in *Theileria*-infected cells. Human DET1 (de-etiolated 1) is a component of the Cul4A-DDB1 ubiquitin ligase complex and was shown to promote the ubiquitination and degradation of the proto-oncogenic transcription factor c-Jun [39]. CUL4-based E3 ligases have been shown to act in tumour suppression, but the DET1/c-Jun link has not been clearly placed in a tumorigenic context or in infection models. Our results show that miR-155 can activate c-Jun and AP-1 in our cells by targeting DET1 and inhibiting its translation. DET1 has also been implicated in transcriptional repression in plants [41]. Here, we showed that the miR-155 effects in DET1 levels led to changes in c-Jun ubiquitination and stabilization without affecting c-Jun transcriptional control. These results explain the elevated c-Jun levels observed in TBL3 cells despite relatively low JNK activity [20]. Furthermore, as the miR-155 binding site is highly conserved across species, it is likely that a similar loop could function in human cancers. Indeed, previous studies of miR-155 in EBV-transformation indicated an enrichment for induced genes with AP-1 binding sites in their promoters [30]. It is worth noting that there is an emerging role of miRNAs as regulators of protein turnover by targeting ubiquitinating proteins. For example, miR-137 targets the mind bomb-1 ubiquitin ligase in neuronal maturation [43] and miR-223 targets the Fbw7 component of the SCF ubiquitin ligase complex [44]. We did not find evidence for changes in the expression of these two miRNAs upon *Theileria* infection. It is also possible that miRNA targeting of ubiquitination machinery may contribute to other aspects of *Theileria*-induced host signaling, such as effects on p53 and NFκB pathways [16], [45].

Overexpression of miR-155 has been functionally linked to tumorigenesis and inflammation in animal models [31], [32], [34]. Moreover, miR-155 appears to be commonly de-regulated by a wide range of infectious agents, including bacteria and viruses [27], [30], [36], [37]. Recent studies have documented the existence of feedback mechanisms between microRNAs and their transcriptional regulators and these autoregulatory loops likely play important roles to balance the state of microRNAs and their protein targets [46]. The regulatory circuit that we have uncovered is unusual in that it involves two negative regulators; one involving miR inhibition of protein translation and the other involving ubiquitin-dependent protein degradation. Each of these steps may be of therapeutic value in attempts to block the oncomiR addiction state. Furthermore, this study highlights the critical role of microRNA pathway function in the parasite-host relationship. Thus, our results place miR-155 at an exciting crossroads between parasitology, regulatory circuits and transformation.

## Materials and Methods

### Parasite-infected cell lines and Buparvaquone treatment

The TBL3 cell line was derived from *in vitro* infection of the spontaneous bovine-B lymphosarcoma cell line, BL3, with Hissar stock of *T. annulata*. The culture conditions and B cell characteristics of these two cell lines have been described previously [47]. The macrophage cell line Thei was isolated from *T. annulata* naturally infected cow. Cell were provided by G Langsley (Paris, France). All cell lines were cultured in RPMI 1640 (Gibco Ltd., Paisley, UK), supplemented with 10% heat-inactivated Fetal calf serum, 4 mM L-Glutamine, 25 mM HEPES, 10 µM β-mercaptoethanol and 100 µg/ml penicillin/streptomycin in a humidified 5% CO_2_ atmosphere at 37°C. For all experiments, cells were seeded at a density of 3×10^5^ cells/ml and exponentially growing cells harvested. The number of cells and viability, as judged by the trypan blue dye exclusion test, were determined by counting the cells in a Mallassen chamber. The anti-parasite drug Buparvaquone (BW720c) [48] was used at 100 ng/ml, as described previously [49] for 64 hours (TBL3 and BL3 cell lines) or 72 hours (Thei cells). BW720c has no effect on the growth of mammalian cells [48].

### RNA preparation and Reverse Transcriptase Quantitative PCR

For isolation of long (>200 nt) and small (<200 nt) cellular RNA, 5×10^6^ cells, which had been cultured in the absence or presence of the indicated agents, were harvested and RNAs were prepared with the Nucleospin miRNA kit (Machery Nagel, Hoerdt, France) according to the manufacturer's instructions. The quality and quantity of the resulting RNAs were determined using a Nanodrop spectrophotometer. Oligonucleotides were designed (Supplementary Table S2) and first-strand cDNA was reverse transcribed from 1 µg long RNA using random primers and VILO Superscript III (Invitrogen, Carlsbad, CA, USA) ; and 10 ng small RNA using TaqMan probes for miR-155 and U6 and TaqMan microRNA reverse transcription kit (Applied Biosystems, Foster City, CA, USA). The cDNA was diluted 1∶10 for detection of all transcripts. Quantitative PCR analyses of miRNAs and mRNAs were performed using Taqman miRNA expression or SYBR green, respectively (Applied Biosystems, Foster City, CA, USA) assays according to the manufacturer's protocols in the ABI 7500 real-time PCR system (Applied Biosystems, Foster City, CA, USA). Bovine β-actin and B2M (long RNA) or RNU6B (miRNA) were used as endogenous controls for normalization. The detection of single products was verified by dissociation curve analysis. Relative quantities of mRNA and miRNA were analyzed by using the delta Ct method. qRT-PCR was repeated for three independent biological replicates of infected cells and experimental duplicates.

### Immunoblot analysis

Cells were sonicated in 2× Laemmli buffer : 15 secs ON/30 secs OFF for 5 mins. Proteins extracts were resolved on 10.5% acrylamide/bis acrylamide SDS-PAGE gels and transferred to nitrocellulose membranes (Thermo Fisher Scientific, Waltham, USA) in transfer buffer. Protein transfer was assessed by Ponceau-red staining. Membranes were blocked in Tris-buffered saline pH 7.4 containing 0.05% Tween-20 and 5% milk for 1 hour at room temperature. Incubations with primary antibodies were carried out at 4°C overnight using antibody dilutions as recommended by the manufacturer in Tris-buffered saline pH 7.4, 0.05% Tween-20 and 5% milk. Following 1 hour of incubation with goat-anti-rabbit peroxidase-conjugated antibody (Sigma, St. Louis, MO, USA) at room temperature, proteins were detected by the chemiluminescence method (Thermo Fisher Scientific, USA) according to the manufacturer's instructions. Antibodies used in immunoblotting were as follows: Rabbit anti-DET1 (Abcam, Cambridge, UK. Ref: ab75918), Rabbit Anti-c-Jun (Santa Cruz Biotechnology, CA, USA. Ref: sc1694), Mouse Anti-αTubulin (Sigma, Ref: T9026), Rabbit Anti-Active Caspase 3 (Sigma, Ref: C8487), Mouse Anti-Ubiquitin (P4D1) (Santa Cruz Biotechnology, CA, USA. Ref: sc-8017). c-Jun or DET1/Tubulin ratios were calculated after western blot signal quantification with the Plot Lanes Analysis tool of Image J software (NIH).

### Reporter constructs and plasmids

Most Luciferase reporters were constructs previously described [30]. Briefly, the *BIC* promoter extends from −1556 to +166 and was cloned into pGL3basic (Promega, Madison, WI, USA). AP1 and NFκB point mutations were generated using the QuikChange II site-directed mutagenesis kit (Stratagene) as previously described [30]. Wild-type or mutated 3′UTRs were cloned downstream from the Luciferase gene in the pMIR-REPORT plasmid (Applied Biosystems, USA). DET1 reporter contains most of the 3′ UTR (131–425 of UTR). For mutant DET1 (previously unpublished), the miR-155 binding sites were mutated by exchanging 4 bases within the seed sequence. Mutations were generated using QuikChange II site-directed mutagenesis kit (Stratagene). TP53INP1 reporter contains 400 bases of 3′ UTR sequences from 312–712. JARID2 reporter contains the 3′ UTR (sequences from 9 to 1214). miR155 (Gene ID: 406947) sequences were cloned downstream from the GFP gene in pMSCV-puro-GFP as previously described [30]. For the miR-155 Sponge plasmid, 10 inverted copies of a bulge forming anti-sense miR-155 sequences (5′- ACTAGTACCCCTATCAGTCTAGCATTAAGGGTTTACCCCTATCAATGTAGCATTAACACAGAACCCCTATCAGAGTAGCATTAAGAGCAGACCCCTATCATTGTAGCATTAAGTGGAAACCCCTATCAACTTAGCATTAACCTTGAACCCCTATCAAGGTAGCATTAAGGACCAACCCCTATCATACTAGCATTAACGAGATACCCCTATCATCTTAGCATTAACCAGGTACCCCTATCAGGATAGCATTAAGGTGCTACCCCTATCAGCCTAGCATTAATCTAGA-3′) were cloned downstream from the GFP gene in the vector, pMSCV-puro-GFP-miRcntl between the NotI and EcoRI sites, as previously described [50]. The complete sequences and maps of this and other plasmids can be found at www.flemingtonlab.com. Unique/different short spacer regions are included between inverted miR-155 sequences to prevent the formation of exact repeats (to prevent recombination events). A Flag-tagged c-Jun dominant negative (DN) mutant Δ169 cDNA was cloned into pCDNA1, and c-Jun cDNA into pHT108. These two plasmids were kindly provided by G Langsley (Paris, France). The DET1 gene was targeted using siRNA oligonucleotides against the bovine DET1 sequence (AAAACCACCTGTTTATCAAGT) and results were compared to transfection with a non-relevant ‘scrambled’ control siRNA.

### Transfections

Thei cells were transfected using Nucleofector kit solution L according to the manufacturer's instructions using Amaxa Nucleofector II device (program T-20) (Lonza, Basel, Switzerland). BL3 and TBL3 cells were transfected using Neon Transfection kit (Invitrogen, CA, USA). Cells were seeded in 24-well plates for 24 h, and then transfected or co-transfected with 1 µg of the indicated constructs for 36 h.

### Luciferase reporter assay

The cells are transfected with luciferase constructs with or without miR-155, Sponge, c-Jun, DN c-Jun and with or without Buparvaquone treatment. Efficiencies of transfections were normalized to Renilla activity by co-transfection of a pRL-TK *Renilla* reporter plasmid (Promega Ref: E6241). Luciferase assay was performed 36 h after transfection using the Dual-Luciferase Reporter Assay System (Promega, Ref: E1980) in a microplate luminometer. Relative luminescence was represented as the ratio firefly/renilla luminescence and then compared with the corresponding empty vector as a control.

### Flow-cytometric analysis of cell-cycle

Cells were collected and fixed in 3.7% Formaldehyde for 40 min on ice and then cold Ethanol 70% for 15 min at 4°C. The cells were stained with propidium iodide (50 µg/ml) and RNase A (1 µg/ml) for 15 min at room temperature. Flow cytometric analysis was done using a FACScan instrument (Becton Dickinson, Mountain View, CA, USA) and CellQuest software.

### miRNAs microarray analysis

Total RNAs was prepared using QIAGEN RNeasy mini kit (Qiagen, Germantown, MD, USA) according to the manufacturer's protocol from two separate Buparvaquone-treated samples of TBL3 cells. The quality and quantity of the RNA samples were assessed using the Experion machine (Bio-Rad Laboratories, USA). The microRNA expression profiling service from Dharmacon (Thermo Fisher Scientific) performed the miRNA microarray analysis.

### Data and statistical analysis

SPSS 19.0 program (SPSS Inc. Chicago, IL, USA) was used for statistics. The results presented in all the figures represent the mean ± SEM of at least three independent experiments. Statistical analysis was performed using the paired-samples t-test to analyze the significant difference between the control and treatment groups. p values of <0.05 were considered statistically significant and are indicated with asterisks.

### Soft agar colony forming assay

A two-layer soft agar culture system was used. Cell counts were performed on a Malassen chamber. A total of 20,000 cells were plated in a volume of 1.5 ml (0.7% SeaKem ME Agarose (Lonza, Ref: 50011)+2× DMEM 20% Fetal calf Serum) over 1.5-ml base layer (1% SeaKem ME Agarose +2× DMEM 20% Fetal calf Serum) in 6-well plates. Cultures were incubated in humidified 37°C incubators with an atmosphere of 5% CO_2_ in air, and control plates were monitored for growth using a microscope. At the time of maximum colony formation (10 days in culture), final colony numbers were counted manually after fixation with 0.005% Cristal Violet (Sigma, Ref: C3886).

### Immunoprecipitation

Cells were treated for 3 h at 37°C with 20 uM MG132 and lysed 10 min on ice in the following buffer: 150 mM NaCl, 1% Nonidet P-40, 0,5% Deoxycholate, 0,1% SDS, 50 mM Tris-HCl pH 7,5, 20 mM NEM, 5 mM Iodoacetamide, 100 uM MG132, 2 mg/mL Pefabloc SC (Roche) and 5 ug/mL each Aprotinin, Leupeptin, Pepstatin. Equal amounts of total cellular proteins were immunoprecipitated with Rabbit Anti-c-Jun (E254) (Abcam, Cambridge, UK. Ref: ab32137) coupled to protein G sepharose beads (Sigma, Ref: P3296) for 90 min at 4°C. After three washes, immunoprecipitated proteins were eluted in Laemmli sample buffer at 95°C for 5 min, resolved by SDS-PAGE and analyzed by western blot using the indicated antibodies. Immunoprecipitation was repeated for three independent biological replicates.

### Cycloheximide chase assay

Transient transfected cells with indicated constructs were treated 30, 60 or 120 min with 100 µg/mL Cycloheximide, 36 h post transfection. Cells were lysed in Laemmli sample buffer, resolved by SDS-PAGE and analyzed by western blot using the indicated antibodies. Relative quantification indicates the c-Jun/Tubulin ratios calculated with Image J software (NIH) and c-Jun levels at time 0 was set as 1. Cycloheximide chase experiments were repeated for three independent biological replicates.

### MG132 treatment

Transient transfected TBL3 cells with miR-155 Sponge were treated 3 h with 20 µM MG132. Cells were lysed in Laemmli sample buffer, resolved by SDS-PAGE and analyzed by Western blot using the indicated antibodies. Relative quantification indicates the c-Jun/Tubulin ratios calculated with Image J software (NIH). MG132 treatment was repeated for three independent biological replicates.

### Ki67 immunofluorescence

Cells were plated on Fibronectin coated slides and then fixed in PBS 3.7% Formaldehyde for 15 min at room temperature. Slides were rinsed in PBS and permeabilized with PBS 0.2% Triton X-100 for 5 min and then blocked for 30 min with PBS 1% SVF and 1% BSA to prevent non-specific staining. The slides were incubated with Mouse monoclonal anti-Ki67 (1∶50, Abcam Cambridge, UK. Ref :ab10913-1) in PBS 1% SVF and 1% BSA at room temperature for 40 min. After washing in PBS 0.2% Tween, the slides were incubated with Cy2 AffinyPure anti-mouse IgG (1∶5000, Jackson Immunology, USA. Ref :715-225-150) for 30 min. Slides were subsequently washed in PBS 0.2% Tween, mounted on slides and coverslipped with ProLong Gold Antifade Reagent with Dapi (Invitrogen, USA. Ref : P-36931). Images of immunofluorescence staining were photographed with a camera attached to a fluorescent microscope (Leica Inverted 6000) and percentage of Ki67 positive cells was calculated. This staining was repeated for three independent biological replicates.

## Supporting Information

Figure S1 **The effect of Buparvaquone treatment on the growth and survival of infected cell lines.** **(A)** The parasite-infected THEI cells were grown in the presence or absence of Buparvaquone (+Bup) and cell numbers were monitored by counting live cells followed by trypan blue exclusion (left panel). Representation of flow cytometry analysis indicating the induction of apoptosis (sub-G1 population) and growth arrest (G1 population) of THEI cells following treatment with Buparvaquone (open histograms - right panel) (average ± sd, n = 3). *p<0.05, **p<0.01 **(B)** BL3 and TBL3 were treated with Buparvaquone for 64 h and cycling cells were measured by immunofluorescence using an anti-Ki67 antibody. Quantification of Ki67-positive cells was monitored by fluorescence microscopy (average ± sd, n = 3). *p<0.05, **p<0.01
(TIF)

Figure S2 **Analysis of putative miR155 oncomiR target genes.** **(A)** Conservation of the seed sequences of miR155 in the 3′UTR of predicted target genes, *DET1*, *JARID2* and *TP53INP1*, identified by computational analysis, in human, cow, mouse and chicken sequences. **(B)** Luciferase reporters containing the 3′UTR of DET1, JARID2 and TP53INP1 were compared with a control Luciferease reporter (pMIR-REP-dCMV), demonstrating that Buparvaquone (+Bup) induced Luciferase activity in parasitized THEI cells (average ± sd, n = 3). *p<0.05, **p<0.01 **(C)** Buparvaquone treatment had no effect on the mRNA levels of *DET1* or *TP53INP1* in the three different cell lines, as assessed by qPCR analysis for the bovine genes. Transcript levels in untreated cells are shown relative to the control and normalized against β-actin and β2M mRNA (average ± sd, n = 3).
(TIF)

Figure S3 **miR155 does not affect c-Jun transcription.** **(A)** The overexpression of miR155 or siDET1 or the miR-155 Sponge had no effect on c-Jun mRNA levels in BL3 cells (left) or TBL3 cells (right), as assessed by qPCR analysis. Transcript levels are shown relative to the control plasmids or scrambled siControls (gray bars) and normalized against β-actin and β2M mRNA (average ± sd, n = 3). **(B)** The miR155 Sponge had no significant effect on c-Jun mRNA levels in TBL3 cells treated or not with MG132, as assessed by qPCR analysis. Transcript levels are shown relative to the control plasmid and normalized against β-actin and B2M mRNA (average ± sd, n = 3). **(C)** miR155 inhibition in TBL3 cells increased c-Jun ubiquitination. Transfected TBL3 cells were treated with MG132 for 3 h, followed by immunoprecipitation of endogenous c-Jun protein and immunoblot analysis with antibodies against Ubiquitin or c-Jun (average ± sd, n = 3).
(TIF)

Table S1 **Summary of additional microRNAs downregulated more than two-fold (Log2) upon Buparvaquone treatment in TBL3 or Thei cells. The table shows the known functions and known target genes and references.**
(PPT)

Table S2 **Oligonucleotide primer sequences used to analyze the expression of genes.** List of oligonucleotide sequences (sense and antisense) used for PCR analysis.
(PPT)
