# Supplementary figures and images for: OncomiR Addiction Is Generated by a miR-155 Feedback Loop in Theileria-Transformed Leukocytes
Source: PLoS Pathog. 2013 Apr 18;9(4):e1003222. doi: 10.1371/journal.ppat.1003222 (PMC3630095; doi:10.1371/journal.ppat.1003222)

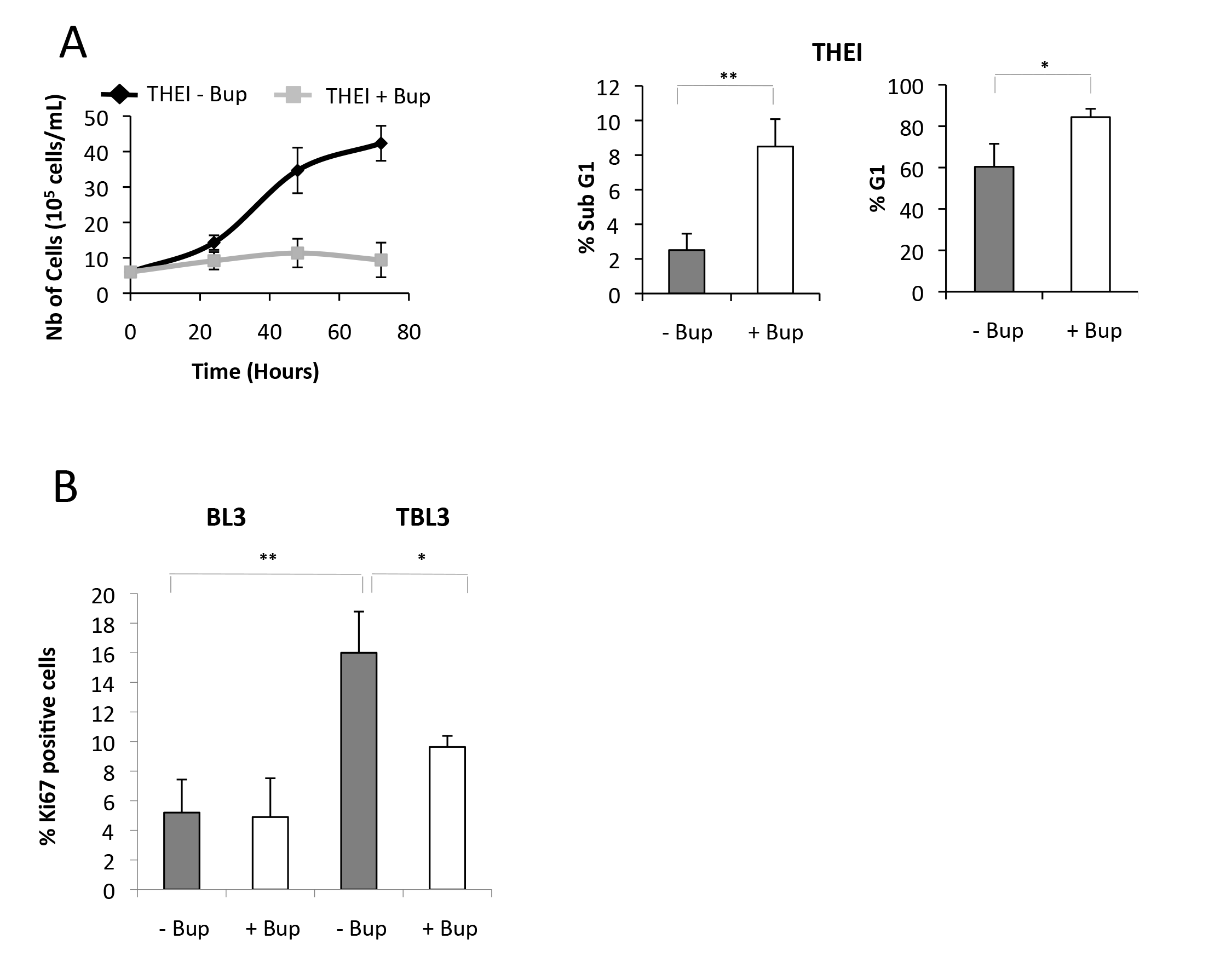

Supplement: Figure S1 — The effect of Buparvaquone treatment on the growth and survival of infected cell lines. (A) The parasite-infected THEI cells were grown in the presence or absence of Buparvaquone (+Bup) and cell numbers were monitored by counting live cells followed by trypan blue exclusion (left panel). Representation of flow cytometry analysis indicating the induction of apoptosis (sub-G1 population) and growth arrest (G1 population) of THEI cells following treatment with Buparvaquone (open histograms - right panel) (average ± sd, n = 3). *p<0.05, **p<0.01 (B) BL3 and TBL3 were treated with Buparvaquone for 64 h and cycling cells were measured by immunofluorescence using an anti-Ki67 antibody. Quantification of Ki67-positive cells was monitored by fluorescence microscopy (average ± sd, n = 3). *p<0.05, **p<0.01 (TIF) [file ppat.1003222.s001.tif]

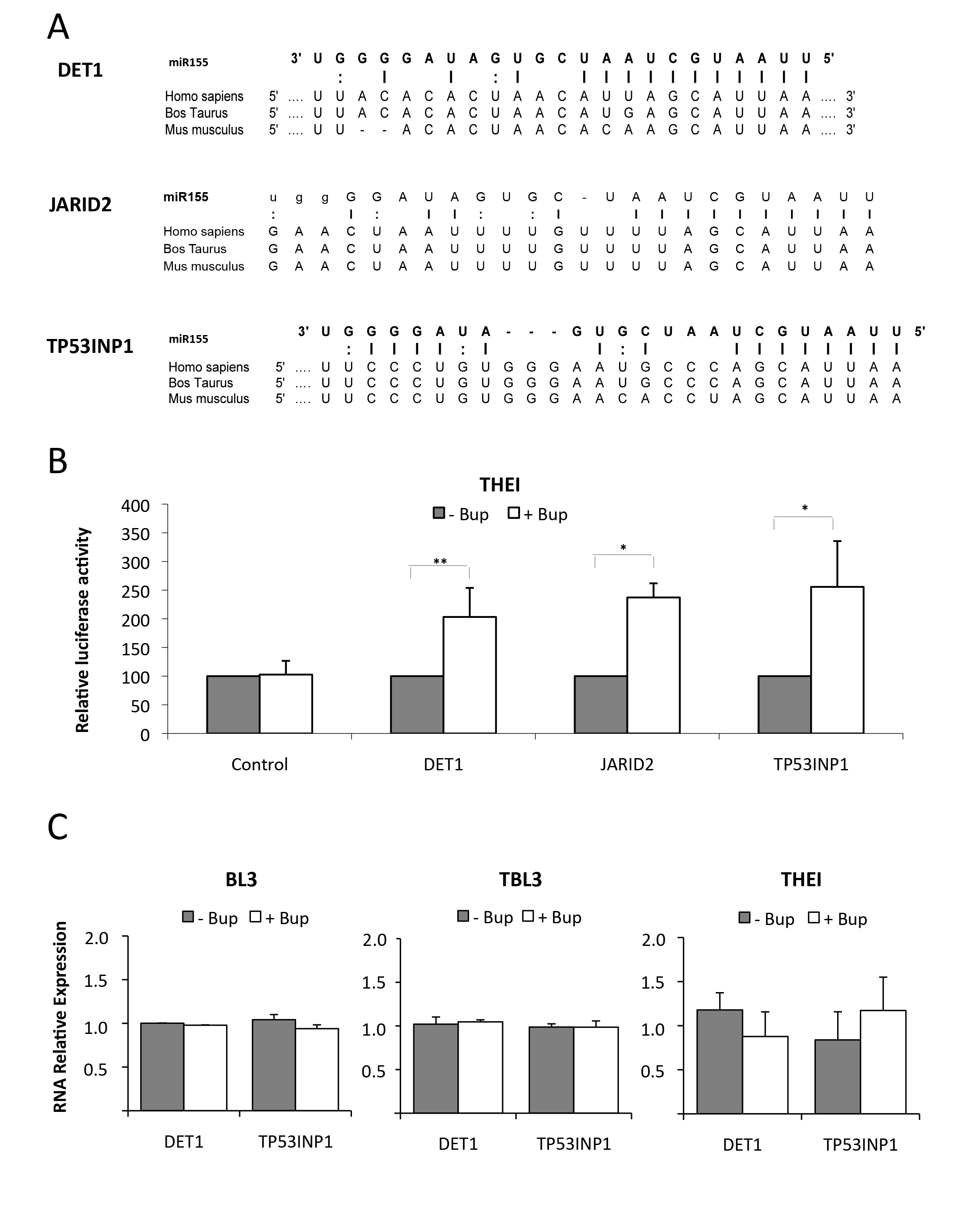

Supplement: Figure S2 — Analysis of putative miR155 oncomiR target genes. (A) Conservation of the seed sequences of miR155 in the 3′UTR of predicted target genes, DET1, JARID2 and TP53INP1, identified by computational analysis, in human, cow, mouse and chicken sequences. (B) Luciferase reporters containing the 3′UTR of DET1, JARID2 and TP53INP1 were compared with a control Luciferease reporter (pMIR-REP-dCMV), demonstrating that Buparvaquone (+Bup) induced Luciferase activity in parasitized THEI cells (average ± sd, n = 3). *p<0.05, **p<0.01 (C) Buparvaquone treatment had no effect on the mRNA levels of DET1 or TP53INP1 in the three different cell lines, as assessed by qPCR analysis for the bovine genes. Transcript levels in untreated cells are shown relative to the control and normalized against β-actin and β2M mRNA (average ± sd, n = 3). (TIF) [file ppat.1003222.s002.tif]

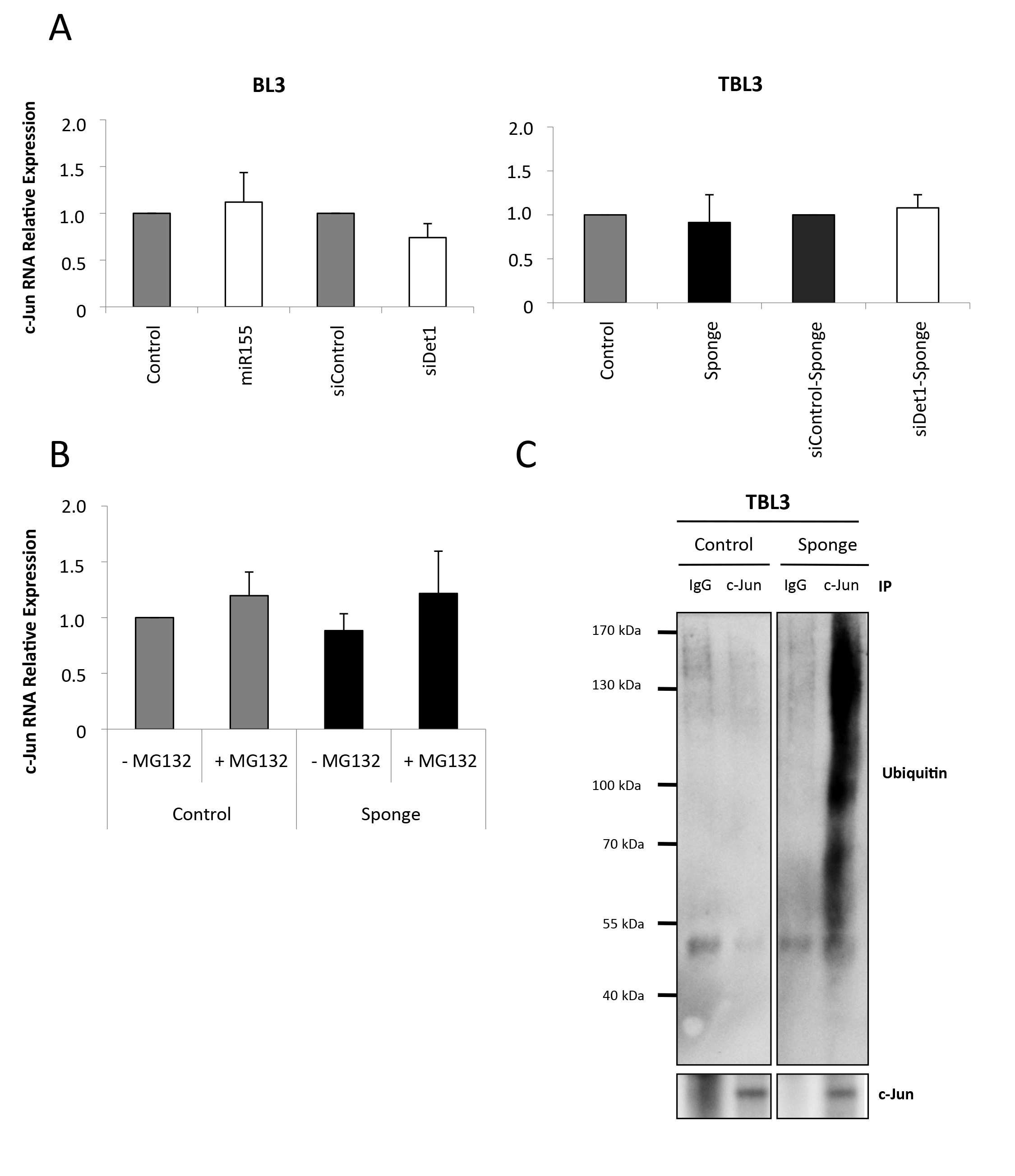

Supplement: Figure S3 — miR155 does not affect c-Jun transcription. (A) The overexpression of miR155 or siDET1 or the miR-155 Sponge had no effect on c-Jun mRNA levels in BL3 cells (left) or TBL3 cells (right), as assessed by qPCR analysis. Transcript levels are shown relative to the control plasmids or scrambled siControls (gray bars) and normalized against β-actin and β2M mRNA (average ± sd, n = 3). (B) The miR155 Sponge had no significant effect on c-Jun mRNA levels in TBL3 cells treated or not with MG132, as assessed by qPCR analysis. Transcript levels are shown relative to the control plasmid and normalized against β-actin and B2M mRNA (average ± sd, n = 3). (C) miR155 inhibition in TBL3 cells increased c-Jun ubiquitination. Transfected TBL3 cells were treated with MG132 for 3 h, followed by immunoprecipitation of endogenous c-Jun protein and immunoblot analysis with antibodies against Ubiquitin or c-Jun (average ± sd, n = 3). (TIF) [file ppat.1003222.s003.tif]
