# Supplementary material for: OncomiR Addiction Is Generated by a miR-155 Feedback Loop in Theileria-Transformed Leukocytes
Source: PLoS Pathog. 2013 Apr 18;9(4):e1003222. doi: 10.1371/journal.ppat.1003222 (PMC3630095; doi:10.1371/journal.ppat.1003222)
Supplement: Table S1 — Summary of additional microRNAs downregulated more than two-fold (Log2) upon Buparvaquone treatment in TBL3 or Thei cells. The table shows the known functions and known target genes and references. (PPT) [file ppat.1003222.s004.ppt]

## Slide 1
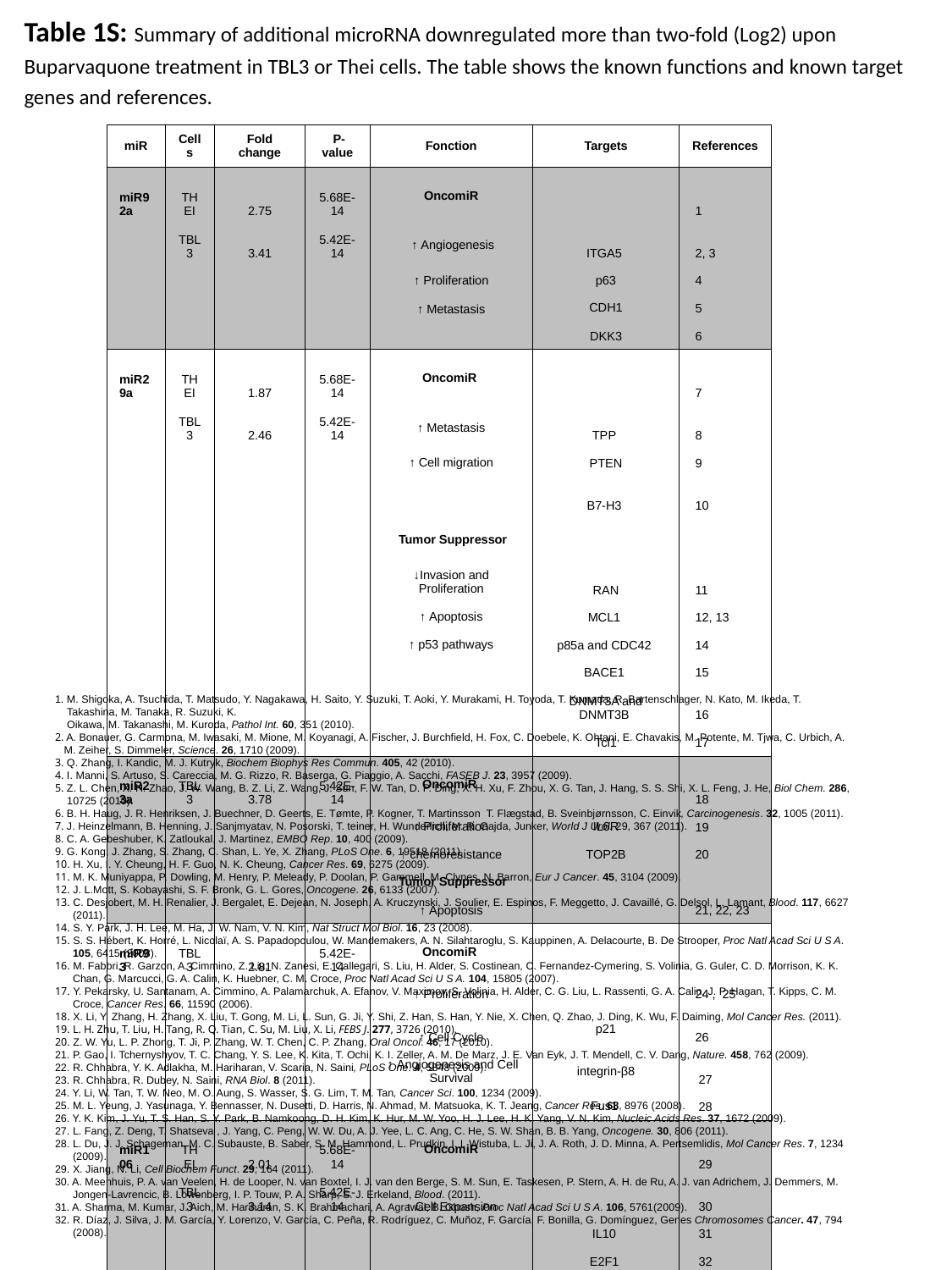

Table 1S: Summary of additional microRNA downregulated more than two-fold (Log2) upon
Buparvaquone treatment in TBL3 or Thei cells. The table shows the known functions and known target
genes and references.
| miR | Cells | Fold change | P-value | Fonction | Targets | References |
| --- | --- | --- | --- | --- | --- | --- |
| miR92a | THEI | 2.75 | 5.68E-14 | OncomiR | | 1 |
| | TBL3 | 3.41 | 5.42E-14 | ↑ Angiogenesis | ITGA5 | 2, 3 |
| | | | | ↑ Proliferation | p63 | 4 |
| | | | | ↑ Metastasis | CDH1 | 5 |
| | | | | | DKK3 | 6 |
| miR29a | THEI | 1.87 | 5.68E-14 | OncomiR | | 7 |
| | TBL3 | 2.46 | 5.42E-14 | ↑ Metastasis | TPP | 8 |
| | | | | ↑ Cell migration | PTEN | 9 |
| | | | | | B7-H3 | 10 |
| | | | | Tumor Suppressor | | |
| | | | | ↓Invasion and Proliferation | RAN | 11 |
| | | | | ↑ Apoptosis | MCL1 | 12, 13 |
| | | | | ↑ p53 pathways | p85a and CDC42 | 14 |
| | | | | | BACE1 | 15 |
| | | | | | DNMT3A and DNMT3B | 16 |
| | | | | | Tcl1 | 17 |
| miR23a | TBL3 | 3.78 | 5.42E-14 | OncomiR | | 18 |
| | | | | ↑ Proliferation | IL6R | 19 |
| | | | | ↑ chemoresistance | TOP2B | 20 |
| | | | | Tumor Suppressor | | |
| | | | | ↑ Apoptosis | | 21, 22, 23 |
| miR93 | TBL3 | 2.81 | 5.42E-14 | OncomiR | | |
| | | | | ↑ Proliferation | | 24 , 25 |
| | | | | ↑ Cell Cycle | p21 | 26 |
| | | | | ↑ Angiogenesis and Cell Survival | integrin-β8 | 27 |
| | | | | | Fus1 | 28 |
| miR106 | THEI | 2.01 | 5.68E-14 | OncomiR | | 29 |
| | TBL3 | 3.14 | 5.42E-14 | ↑ Cell Expansion | | 30 |
| | | | | | IL10 | 31 |
| | | | | | E2F1 | 32 |
1. M. Shigoka, A. Tsuchida, T. Matsudo, Y. Nagakawa, H. Saito, Y. Suzuki, T. Aoki, Y. Murakami, H. Toyoda, T. Kumada, R. Bartenschlager, N. Kato, M. Ikeda, T.
 Takashina, M. Tanaka, R. Suzuki, K.
 Oikawa, M. Takanashi, M. Kuroda, Pathol Int. 60, 351 (2010).
2. A. Bonauer, G. Carmona, M. Iwasaki, M. Mione, M. Koyanagi, A. Fischer, J. Burchfield, H. Fox, C. Doebele, K. Ohtani, E. Chavakis, M. Potente, M. Tjwa, C. Urbich, A.
 M. Zeiher, S. Dimmeler, Science. 26, 1710 (2009).
3. Q. Zhang, I. Kandic, M. J. Kutryk, Biochem Biophys Res Commun. 405, 42 (2010).
4. I. Manni, S. Artuso, S. Careccia, M. G. Rizzo, R. Baserga, G. Piaggio, A. Sacchi, FASEB J. 23, 3957 (2009).
5. Z. L. Chen, X. H. Zhao, J. W. Wang, B. Z. Li, Z. Wang, J. Sun, F. W. Tan, D. P. Ding, X. H. Xu, F. Zhou, X. G. Tan, J. Hang, S. S. Shi, X. L. Feng, J. He, Biol Chem. 286,
 10725 (2010).
6. B. H. Haug, J. R. Henriksen, J. Buechner, D. Geerts, E. Tømte, P. Kogner, T. Martinsson T. Flægstad, B. Sveinbjørnsson, C. Einvik, Carcinogenesis. 32, 1005 (2011).
7. J. Heinzelmann, B. Henning, J. Sanjmyatav, N. Posorski, T. teiner, H. Wunderlich, M. R. Gajda, Junker, World J Urol. 29, 367 (2011).
8. C. A. Gebeshuber, K. Zatloukal, J. Martinez, EMBO Rep. 10, 400 (2009).
9. G. Kong, J. Zhang, S. Zhang, C. Shan, L. Ye, X. Zhang, PLoS One. 6, 19518 (2011).
10. H. Xu, I. Y. Cheung, H. F. Guo, N. K. Cheung, Cancer Res. 69, 6275 (2009).
11. M. K. Muniyappa, P. Dowling, M. Henry, P. Meleady, P. Doolan, P. Gammell, M. Clynes, N. Barron, Eur J Cancer. 45, 3104 (2009).
12. J. L.Mott, S. Kobayashi, S. F. Bronk, G. L. Gores, Oncogene. 26, 6133 (2007).
13. C. Desjobert, M. H. Renalier, J. Bergalet, E. Dejean, N. Joseph, A. Kruczynski, J. Soulier, E. Espinos, F. Meggetto, J. Cavaillé, G. Delsol, L. Lamant, Blood. 117, 6627
 (2011).
14. S. Y. Park, J. H. Lee, M. Ha, J. W. Nam, V. N. Kim, Nat Struct Mol Biol. 16, 23 (2008).
15. S. S. Hébert, K. Horré, L. Nicolaï, A. S. Papadopoulou, W. Mandemakers, A. N. Silahtaroglu, S. Kauppinen, A. Delacourte, B. De Strooper, Proc Natl Acad Sci U S A.
 105, 6415 (2008).
16. M. Fabbri, R. Garzon, A. Cimmino, Z. Liu, N. Zanesi, E. Callegari, S. Liu, H. Alder, S. Costinean, C. Fernandez-Cymering, S. Volinia, G. Guler, C. D. Morrison, K. K.
 Chan, G. Marcucci, G. A. Calin, K. Huebner, C. M. Croce, Proc Natl Acad Sci U S A. 104, 15805 (2007).
17. Y. Pekarsky, U. Santanam, A. Cimmino, A. Palamarchuk, A. Efanov, V. Maximov, S. Volinia, H. Alder, C. G. Liu, L. Rassenti, G. A. Calin, J. P. Hagan, T. Kipps, C. M.
 Croce, Cancer Res. 66, 11590 (2006).
18. X. Li, Y. Zhang, H. Zhang, X. Liu, T. Gong, M. Li, L. Sun, G. Ji, Y. Shi, Z. Han, S. Han, Y. Nie, X. Chen, Q. Zhao, J. Ding, K. Wu, F. Daiming, Mol Cancer Res. (2011).
19. L. H. Zhu, T. Liu, H. Tang, R. Q. Tian, C. Su, M. Liu, X. Li, FEBS J. 277, 3726 (2010).
20. Z. W. Yu, L. P. Zhong, T. Ji, P. Zhang, W. T. Chen, C. P. Zhang, Oral Oncol. 46, 17 (2010).
21. P. Gao, I. Tchernyshyov, T. C. Chang, Y. S. Lee, K. Kita, T. Ochi, K. I. Zeller, A. M. De Marz, J. E. Van Eyk, J. T. Mendell, C. V. Dang, Nature. 458, 762 (2009).
22. R. Chhabra, Y. K. Adlakha, M. Hariharan, V. Scaria, N. Saini, PLoS One. 4, 5848 (2009).
23. R. Chhabra, R. Dubey, N. Saini, RNA Biol. 8 (2011).
24. Y. Li, W. Tan, T. W. Neo, M. O. Aung, S. Wasser, S. G. Lim, T. M. Tan, Cancer Sci. 100, 1234 (2009).
25. M. L. Yeung, J. Yasunaga, Y. Bennasser, N. Dusetti, D. Harris, N. Ahmad, M. Matsuoka, K. T. Jeang, Cancer Res. 68, 8976 (2008).
26. Y. K. Kim, J. Yu, T. S. Han, S. Y. Park, B. Namkoong, D. H. Kim, K. Hur, M. W. Yoo, H. J. Lee, H. K. Yang, V. N. Kim, Nucleic Acids Res. 37, 1672 (2009).
27. L. Fang, Z. Deng, T. Shatseva , J. Yang, C. Peng, W. W. Du, A. J. Yee, L. C. Ang, C. He, S. W. Shan, B. B. Yang, Oncogene. 30, 806 (2011).
28. L. Du, J. J. Schageman, M. C. Subauste, B. Saber, S. M. Hammond, L. Prudkin, I. I. Wistuba, L. Ji, J. A. Roth, J. D. Minna, A. Pertsemlidis, Mol Cancer Res. 7, 1234
 (2009).
29. X. Jiang, N. Li, Cell Biochem Funct. 29, 164 (2011).
30. A. Meenhuis, P. A. van Veelen, H. de Looper, N. van Boxtel, I. J. van den Berge, S. M. Sun, E. Taskesen, P. Stern, A. H. de Ru, A. J. van Adrichem, J. Demmers, M.
 Jongen-Lavrencic, B. Löwenberg, I. P. Touw, P. A. Sharp, S. J. Erkeland, Blood. (2011).
31. A. Sharma, M. Kumar, J. Aich, M. Hariharan, S. K. Brahmachari, A. Agrawal, B. Ghosh, Proc Natl Acad Sci U S A. 106, 5761(2009).
32. R. Díaz, J. Silva, J. M. García, Y. Lorenzo, V. García, C. Peña, R. Rodríguez, C. Muñoz, F. García, F. Bonilla, G. Domínguez, Genes Chromosomes Cancer. 47, 794
 (2008).
