# Supplementary material for: OncomiR Addiction Is Generated by a miR-155 Feedback Loop in Theileria-Transformed Leukocytes
Source: PLoS Pathog. 2013 Apr 18;9(4):e1003222. doi: 10.1371/journal.ppat.1003222 (PMC3630095; doi:10.1371/journal.ppat.1003222)
Supplement: Table S2 — Oligonucleotide primer sequences used to analyze the expression of genes. List of oligonucleotide sequences (sense and antisense) used for PCR analysis. (PPT) [file ppat.1003222.s005.ppt]

## Slide 1
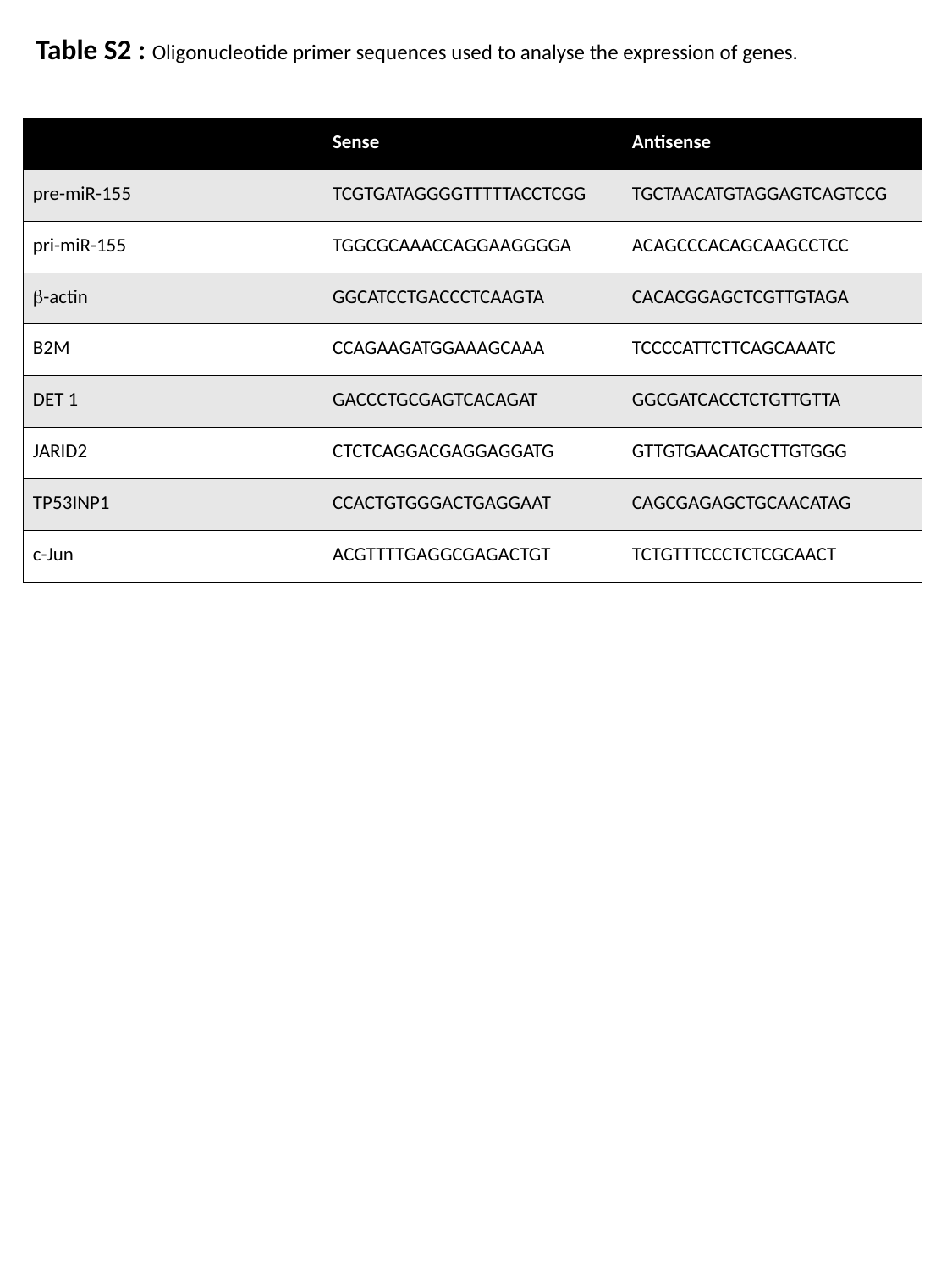

Table S2 : Oligonucleotide primer sequences used to analyse the expression of genes.
| | Sense | Antisense |
| --- | --- | --- |
| pre-miR-155 | TCGTGATAGGGGTTTTTACCTCGG | TGCTAACATGTAGGAGTCAGTCCG |
| pri-miR-155 | TGGCGCAAACCAGGAAGGGGA | ACAGCCCACAGCAAGCCTCC |
| β-actin | GGCATCCTGACCCTCAAGTA | CACACGGAGCTCGTTGTAGA |
| B2M | CCAGAAGATGGAAAGCAAA | TCCCCATTCTTCAGCAAATC |
| DET 1 | GACCCTGCGAGTCACAGAT | GGCGATCACCTCTGTTGTTA |
| JARID2 | CTCTCAGGACGAGGAGGATG | GTTGTGAACATGCTTGTGGG |
| TP53INP1 | CCACTGTGGGACTGAGGAAT | CAGCGAGAGCTGCAACATAG |
| c-Jun | ACGTTTTGAGGCGAGACTGT | TCTGTTTCCCTCTCGCAACT |
